# LSD1 inhibition sustains T cell invigoration with a durable response to PD-1 blockade

**DOI:** 10.1038/s41467-021-27179-7

**Published:** 2021-11-24

**Authors:** Yi Liu, Brian Debo, Mingfeng Li, Zhennan Shi, Wanqiang Sheng, Yang Shi

**Affiliations:** 1 Division of Newborn Medicine and Epigenetics Program, Boston Children’s Hospital, Harvard Medical School, Boston, MA 02115 USA; 2 Ludwig Institute for Cancer Research, University of Oxford, Oxford, OX3 7DQ UK; 3 Department of Neuroscience and Kavli Institute for Neuroscience, Yale School of Medicine, New Haven, CT 06510 USA; 4 Institute of Immunology, and Department of Respiratory Disease of The First Affiliated Hospital, Zhejiang University School of Medicine, Hangzhou, Zhejiang, 310058 China

**Keywords:** Tumour immunology, Epigenetics

## Abstract

Exhausted CD8^+^ T cells are key targets of immune checkpoint blockade therapy and their ineffective reinvigoration limits the durable benefit in some cancer patients. Here, we demonstrate that histone demethylase LSD1 acts to enforce an epigenetic program in progenitor exhausted CD8^+^ T cells to antagonize the TCF1-mediated progenitor maintenance and to promote terminal differentiation. Consequently, genetic perturbation or small molecules targeting LSD1 increases the persistence of the progenitor exhausted CD8^+^ T cells, which provide a sustained source for the proliferative conversion to numerically larger terminally exhausted T cells with tumor-killing cytotoxicity, thereby leading to effective and durable responses to anti-PD1 therapy. Collectively, our findings provide important insights into epigenetic mechanisms that regulate T cell exhaustion and have important implications for durable immunotherapy.

## Abstract (web summary)

Dynamic changes in chromatin landscape affect CD8^+^ T cell phenotype and function in chronic infections and cancer. Here the authors show that targeting the histone demethylase LSD1 increases the persistence of progenitor exhausted CD8^+^ T cells, improving response to immune checkpoint blockade in preclinical cancer models.

## Introduction

Immune checkpoint blockade, exemplified by using PD-1 blocking antibodies, has shown remarkable clinical success^1,2^. However, a majority of cancer patients have not benefited from this therapy to date. Notably, some cancer patients who initially respond to anti-PD-1 therapy develop tumor progression after a period of time in spite of continuous treatment^1,3^, highlighting the importance of improving T cell response to PD-1 blockade. The prolonged stimulation of T cell receptor (TCR) by cognate antigens drives CD8^+^ T cell exhaustion, which is maintained by the interaction between the high-level of PD-1 in T cells and PD-L1 in tumors^2,4,5^. The use of PD-1 blocking antibodies has been shown to abrogate this inhibitory effect and reinvigorate the exhausted T cells^2,4^. However, the molecular mechanisms leading to T cell exhaustion and to what extent exhausted T cells can be reinvigorated remain incompletely understood. Recently, in chronic viral infections and cancer^6–8^, exhausted CD8^+^ T cells have been defined to include at least two distinct subsets - a progenitor subset expressing an intermediate level of PD-1 and the transcription factor TCF1 (PD-1^int^TCF1^+^), and a more differentiated subset lacking TCF1 while expressing a high level of PD-1 (PD-1^hi^TCF1^-^) that contains the terminally exhausted cells^9–13^. The progenitor exhausted CD8^+^ T cells retain higher proliferation capacity and better ability to produce cytokines, and can maintain self-renewal while continuously giving rise to numerically more differentiated cells that have stronger cytotoxicity, but are increasingly prone to apoptosis^2,4^. In line with these properties, the progenitor exhausted CD8^+^ T cells have been reported as the prominent determinant of effective responses to PD-1 blockade^6,8,9,13^. Indeed, in melanoma patients receiving anti-PD-1 treatment, the frequency of intratumoral TCF1^+^ progenitor CD8^+^ T cells is correlated with positive outcomes^7^. Since proliferation of the progenitor exhausted CD8^+^ T cells in response to TCR stimulation and PD-1 blockade progressively leads to their conversion to terminally exhausted phenotype^2^, approaches to maintain or expand the progenitor subset of exhausted CD8^+^ T cells may help sustain anti-tumor response induced by anti-PD-1 therapy. In support of this hypothesis, the duration of response in melanoma patients who respond to anti-PD-1 treatment is positively correlated with the frequency of progenitor exhausted CD8^+^ T cells^6^. Thus, understanding the mechanisms that control progenitor exhausted CD8^+^ T cell generation, maintenance, and differentiation to a terminal exhaustion state is of particular importance for predicting tumor response to anti-PD-1 treatment and for enhancing the treatment efficacy.

Massive changes occur in chromatin landscape when CD8^+^ T cells become exhausted in chronic viral infections and cancer^6,14–17^. Chromatin modifications have the potential to efficiently and stably silence relevant genes to create a barrier, which prevents the reinvigoration of exhausted CD8^+^ T cells in response to PD-1 blockade. Indeed, the de novo DNA methyltransferase, DNMT3A, has been reported to mediate DNA methylation and transcriptional silencing of both cytotoxicity- and self-renewal-related genes in exhausted CD8^+^ T cells, and the methylation status remains largely stable even upon anti-PD-L1 treatment^18^. In addition, histone methyltransferase EZH2 has also been reported to regulate CD8^+^ T cell differentiation and function in viral infections or cancer^19–22^, but the impact of perturbing T cell-intrinsic EZH2 on T cell response to PD-1 blockade remains unclear. While a number of transcription factors, including TOX, T-bet, and Nr4A, have been discovered to regulate T cell exhaustion^23–29^, chromatin regulatory mechanisms in T cell exhaustion have not been fully explored. The identification and characterization of new epigenetic regulators in T cell exhaustion could uncover potential druggable targets for therapeutic intervention to potentiate the effectiveness and sustainability of PD-1 blockade therapy.

In this study, we identify histone demethylase LSD1 as an important modulator of T cell exhaustion in cancer. LSD1 loss in T cells expands the pool size of the progenitor subset of exhausted CD8^+^ T cells in a variety of mouse tumor models. Mechanistically, we demonstrate that LSD1 physically interacts with the long isoform of TCF1 and antagonizes its transcriptional activity. Consequently, LSD1 inhibition augments the transcriptional network controlled by TCF1 essential for maintaining the progenitor phenotype. In response to PD-1 blocking antibodies, the increased pool of progenitor exhausted CD8^+^ T cells caused by LSD1 inhibition provides a sustained source for the conversion to more differentiated T cells with stronger tumor-killing cytotoxicity, which enables a long-lasting response to anti-PD-1 treatment.

## Results

### LSD1 modulates T cell immunity depending on tumor context

**Fig. 1:**
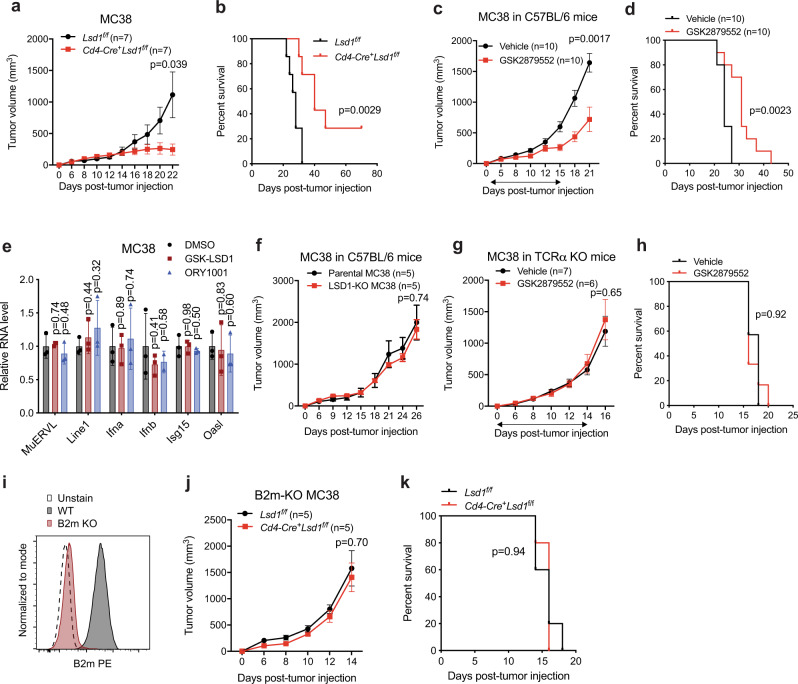
LSD1 inhibition in T cells enhances antitumor immunity in MC38 tumor model. **a**, **b** Tumor growth (**a**) and survival curves (**b**) of *Lsd1*^*f/f*^ and *Cd4-Cre*^*+*^*Lsd1*^*f/f*^ mice subcutaneously inoculated with MC38 tumor cells (*n* = 7 per group). **c**, **d** Tumor growth (**c**) and survival curves (**d**) of wildtype mice inoculated with MC38 tumor cells and treated with LSD1 inhibitor GSK2879552 or vehicle control daily for 2 weeks (arrow line). **e** The real-time qPCR analysis of IFN-related genes in cultured MC38 cells treated with 2 μM GSK-LSD1, 0.5 μM ORY1001 or vehicle control for 5 days (*n* = 3). **f** Tumor growth curves of wildtype mice inoculated with wildtype parental or Lsd1 KO MC38 tumor cells. **g**, **h** Tumor growth (**g**) and survival curves (**h**) of TCRα KO mice inoculated with wildtype MC38 tumor cells and treated with GSK2879552 or vehicle control daily for 2 weeks (arrow line, vehicle group, *n* = 7; GSK2879552 group, *n* = 6). **i** Flow cytometry analysis of B2m expression by WT and B2m KO MC38 cells. **j**, **k** Tumor growth (**j**) and survival curves (**k**) of *Lsd1*^*f/f*^ and *Cd4-Cre*^*+*^*Lsd1*^*f/f*^ mice inoculated with B2m KO MC38 tumor cells (*n* = 5 per group). Data represent two independent experiments and are presented as mean ± SEM (standard error of the mean, **a**, **c**, **f**, **g**, **j**) or mean ± SD (standard deviation, **e**). Sample sizes are as indicated. Statistical significance was determined by two-sided unpaired t test (**a**, **c**, **e**–**g,** **j**) or log-rank test (**b**, **d**, **h**, **k**). Source data are provided as a Source data file.

To investigate the role of LSD1 in T cell immunity in the cancer context, we generated T cell-specific *Lsd1* knockout (*Cd4-Cre*^*+*^*Lsd1*^*f/f*^) mice, which showed largely unaltered αβ T cell development (Supplementary Fig. 1a–c) and displayed an increased central memory phenotype of T cells in peripheral lymphoid organs (Supplementary Fig. 1d–l). We subcutaneously inoculated syngeneic tumors in the *Cd4-Cre*^*+*^*Lsd1*^*f/f*^ and littermate control mice and found that LSD1 depletion in T cells affected tumor growth variably across tumor models. We observed markedly suppressed growth of MC38 colon carcinoma and TRAMP-C2 prostate adenocarcinoma in contrast to significantly accelerated growth of B16/F10 melanoma, when LSD1 was depleted in T cells (Fig. 1a, b and Supplementary Fig. 2a, b). Interestingly, when a foreign antigen Ovalbumin (OVA) was introduced into B16/F10 cells, this tumor growth acceleration was diminished (Supplementary Fig. 2c), suggesting that tumor immunogenicity could be one of the factors that dictate the anti-tumor or pro-tumor effect of T cell-intrinsic LSD1 depletion. For the following study, we mainly focused on the immunogenic MC38 tumor model, and in some instances also the TRAMP-C2 model, to elucidate the T-cell intrinsic role of LSD1 in antitumor T cell immunity.

To complement the genetic finding in MC38 tumor model, we treated wildtype mice with the LSD1 chemical inhibitor, GSK2879552^30^, daily for two weeks and found that this inhibitor acted similarly as *Lsd1* genetic ablation, albeit less profoundly, in suppressing tumor growth (Fig. 1c, d). Since our previous study identified an antitumor immune stimulatory effect caused by LSD1 inhibition-induced type I interferon activation in poorly immunogenic tumor models such as B16/F10 and D4m.3A^31^, we next sought to determine whether this anti-tumor effect of GSK2879552 came from LSD1 inhibition in tumor or immune cells. Unlike B16/F10 melanoma cells, we found no evidence of interferon pathway activation in MC38 cells treated with LSD1 chemical inhibitors such as GSK-LSD1, which is a tool compound of GSK2879552, and ORY1001 (Fig. 1e). Consistently, MC38 tumors with *Lsd1* genetic ablation grew similarly as wildtype tumors when implanted in immunocompetent mice (Fig. 1f). Given that specifically targeting LSD1 in MC38 tumor cells had no significant impact on tumor growth in mice with an intact immune system, these findings point to the importance of GSK2879552 on immune cells. To investigate this further, we used T cell receptor α (TCRα) knockout mice, which are deficient in CD4^+^ and CD8^+^ αβ T cells, and treated tumor-bearing mice with GSK2879552 as aforementioned. We found GSK2879552 failed to suppress MC38 tumor growth in TCRα KO mice (Fig. 1g, h), suggesting that GSK2879552 acts on LSD1 in T cells to augment the antitumor effect, similar to the impact of T cell-specific LSD1 depletion, in certain tumors.

### LSD1-deficient CD8^+^ T cells demonstrate sustained tumor infiltration, which accounts for the extended tumor growth control

Cytotoxic CD8^+^ T cells play a central role in antitumor immunity^32,33^. We hypothesized that CD8^+^ T cells mediated the antitumor effect resulting from T cell-specific LSD1 depletion in MC38 tumor model. To interrogate this possibility, we deleted the *B2m* gene, which encodes an essential component of the major histocompatibility complex class I (MHC-I)^33^, to abolish tumor antigen recognition by CD8^+^ T cells. We confirmed the loss of B2m on MC38 cell surface by flow cytometry (Fig. 1i), and then implanted B2m-deficient tumors into *Cd4-Cre*^*+*^*Lsd1*^*f/f*^ mice or littermate controls to monitor tumor growth. Unlike wildtype tumors (Fig. 1a, b), B2m-deficient MC38 tumors showed indistinguishable growth kinetics in these two groups of mice (Fig. 1j, k), demonstrating that tumor cell recognition by CD8^+^ T cells is essential for the antitumor effect caused by T cell-specific LSD1 depletion. Thus, these results suggest that inhibition of LSD1 in CD8^+^ T cells potentiates their antitumor ability against certain tumors.

**Fig. 2:**
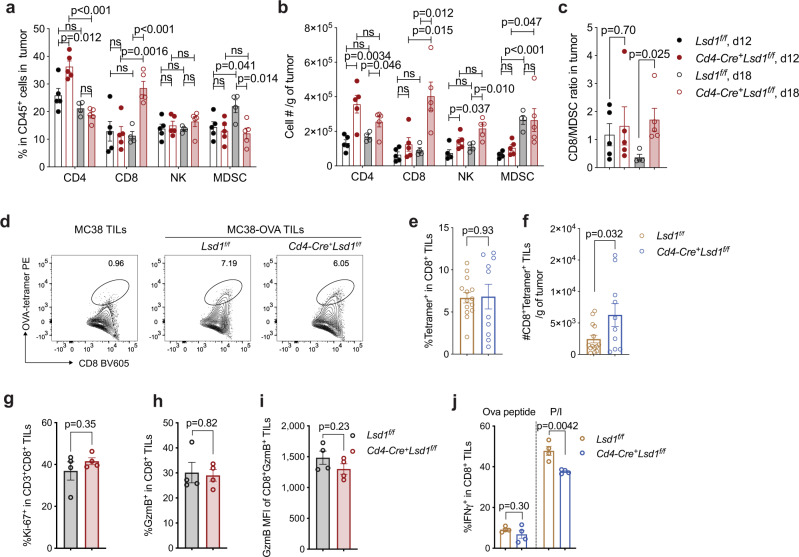
LSD1 depletion results in sustained tumor infiltration of CD8^+^ T cells. **a**, **b** Frequencies of tumor-infiltrating CD3^+^CD4^+^ T cells, CD3^+^CD8^+^ T cells, CD3^-^CD49b^+^ natural killer (NK) cells and CD11b^+^Gr1^+^ myeloid-derived suppressor cells (MDSCs) among gated CD45^+^ leukocytes (**a**) and cell numbers per gram of tumor (**b**) analyzed by flow cytometry on day 12 and 18 after MC38 tumor implantation in *Lsd1*^*f/f*^ and *Cd4-Cre*^*+*^*Lsd1*^*f/f*^ mice (*Lsd1*^*f/f*^ d12 group, *n* = 5; *Cd4-Cre*^*+*^*Lsd1*^*f/f*^ d12 group, *n* = 5; *Lsd1*^*f/f*^ d18 group, *n* = 4; *Cd4-Cre*^*+*^*Lsd1*^*f/f*^ d18 group, *n* = 5). **c** Calculated ratios of intratumoral CD8^+^ T cell number over MDSCs (*n* = 5, 5, 4, or 5, respectively). **d**–**f** Representative flow plots (**d**), percentages (**e**) and cell numbers (**f**) of CD8^+^Tetramer^+^ TILs in implanted MC38-OVA tumors analyzed by flow cytometry on day 18 (*Lsd1*^*f/f*^ group, *n* = 14; *Cd4-Cre*^*+*^*Lsd1*^*f/f*^ group, *n* = 10). **g**–**i** Frequencies of Ki-67-expressing CD8^+^ TILs (**g**), frequencies (**h**) and mean fluorescence intensity (MFI, **i**) of GzmB-expressing CD8^+^ TILs analyzed by flow cytometry on day 18 after MC38 tumor implantation (*n* = 4 per group). **j** IFN-γ expression by MC38-OVA tumor-infiltrating CD8^+^ TILs analyzed by flow cytometry after stimulation with OVA_257-264_ peptides or PMA/Ionomycin (P/I) in the presence of GolgiPlug (*n* = 4 per group). Data represent two independent experiments (**a**–**c**, **g**–**j**) or are pooled from three independent experiments (**e**, **f**) and are presented as mean ± SEM (**a**–**j**). Each dot on the graphs represents an individual mouse. Sample sizes are as indicated. Statistical significance was determined by two-sided unpaired *t* test; ns, not significant (**a**–**j**). Source data are provided as a Source data file.

We next investigated how CD8^+^ T cell immunity in response to tumor growth was regulated by LSD1. The analysis of MC38 tumor growth kinetics suggested that tumor growth can be divided into two stages, an earlier slow growth stage (before day 14) and a later accelerated growth stage (post day 14) (Fig. 1a), which was similarly observed in the TRAMP-C2 tumor model (Supplementary Fig. 2a). Notably, the tumor growth suppression in the *Cd4-Cre*^*+*^*Lsd1*^*f/f*^ mice began to appear only when growth in wildtype mice had already entered the acceleration stage (Fig. 1a). To elucidate the immunologic basis underlying this phenotype, we analyzed tumor-infiltrating leukocytes (TILs) from MC38 tumor-bearing *Cd4-Cre*^*+*^*Lsd1*^*f/f*^ mice and littermate controls at two time points (day 12 and day 18), corresponding to the two stages, which allowed for intra-group longitudinal comparisons as well as inter-group comparisons. In the control *Lsd1*^*f/f*^ mice, antitumor immune populations including CD8^+^ T cells and natural killer (NK) cells did not show noticeable changes in frequencies or cell numbers when tumors grew from day 12 to day 18 (Fig. 2a, b). Instead, the infiltration of myeloid-derived suppressor cells (MDSCs) was significantly elevated (Fig. 2a, b), in association with the accelerated tumor growth. When LSD1 was depleted in T cells, the infiltration of CD8^+^ TILs remained largely unaltered at the earlier growth stage (day 12), but it was drastically increased at the later time point (day 18) (Fig. 2a, b), making the ratio of CD8^+^ TILs cell number over MDSCs significantly higher in the *Cd4-Cre*^*+*^*Lsd1*^*f/f*^ mice than that in the littermate controls (Fig. 2c).

Using Ovalbumin-expressing MC38 tumors (MC38-OVA) and tetramer staining, we found that the percentage of OVA-specific T cells among total CD8^+^ TILs was comparable between these two groups of mice (Fig. 2d, e). Thus, tumor antigen-specific T cells were correspondingly expanded along with the increased total CD8^+^ TILs in response to LSD1 depletion on day 18 (Fig. 2f). In addition, although the total number of CD8^+^ T cells in tumor-draining lymph nodes (TdLNs) was reduced by LSD1 depletion (Supplementary Fig. 3a–d), OVA-specific CD8^+^ T cells in TdLNs were numerically comparable between the two groups of mice carrying MC38-OVA tumors (Supplementary Fig. 3e, f), suggesting CD8^+^ T cell priming in response to the tumor growth is mostly unaffected by LSD1 depletion. As aforementioned, LSD1-competent and -deficient CD8^+^ T cell infiltration was comparable on day 12 (Fig. 2a, b), suggesting that CD8^+^ T cell recruitment to the tumor sites is also unlikely affected by LSD1 depletion, at least at the earlier stage. Thus, we speculated that LSD1 depletion may enhance the local expansion of CD8^+^ TILs after their recruitment into the tumor microenvironment (TME), and this speculation is consistent with the recent studies demonstrating the importance of intratumoral CD8^+^ T cell expansion in tumor control in response to a variety of stimuli^7,8,34^. The effector function of MC38 tumor-infiltrating CD8^+^ T cells, however, appeared largely unaffected as we detected no overt changes in Ki-67 and granzyme B (GzmB) expression and only a modest reduction of IFN-γ expression upon LSD1 depletion (Fig. 2g–j). Taken together, our data suggest that loss of LSD1 in T cells improves the antitumor effect through elevating intratumoral CD8^+^ T cell accumulation, potentially mediated by their local expansion.

### LSD1 loss promotes intratumoral CD8^+^ T cell expansion

**Fig. 3:**
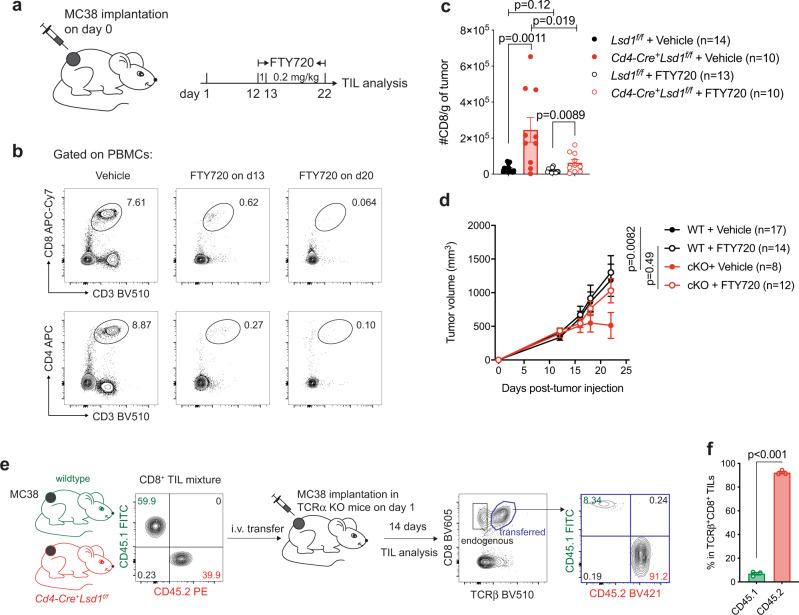
LSD1 loss promotes intratumoral CD8^+^ T cell persistence. **a** Experimental design of administering FTY720 to block T cell egress. **b** Representative flow plots showing frequencies of CD4^+^ and CD8^+^ T cells in PBMCs of mice receiving FTY720 or vehicle. **c** Cell numbers of CD8^+^ TILs on day 22. **d** Tumor growth curves of WT and *Cd4-Cre*^*+*^*Lsd1*^*f/f*^ (cKO) mice implanted with MC38 tumors and treated with FTY720 or vehicle. **e** Experimental design of CD8^+^ TILs co-transfer and assessment of intratumoral persistence. **f** Frequencies of CD45.1^+^ wildtype and CD45.2^+^ LSD1-deficient cells among transferred TCRβ^+^CD8^+^ TILs (*n* = 3 per group). Data are pooled from two experiments and presented as mean ± SEM (**c**, **d**, **f**). Sample sizes are as indicated. Statistical significance was determined by two-sided unpaired *t* test (**c**, **f**) or two-way ANOVA (**d**). Source data are provided as a Source data file.

To examine the local expansion of CD8^+^ T cells in the TME and its regulation by LSD1, we implanted MC38 tumors in *Cd4-Cre*^*+*^*Lsd1*^*f/f*^ mice and littermate controls for 12 days to allow initial T cell infiltration in comparable numbers and then treated those mice with FTY720, a S1P receptor agonist that can block additional T cell recruitment from lymph nodes to tumor sites (Fig. 3a). FTY720 administration effectively blocked T cell egress (Fig. 3b), and brought down the number of CD8^+^ TILs compared with vehicle control in *Cd4-Cre*^*+*^*Lsd1*^*f/f*^ mice (Fig. 3c). In line with this, the inhibition of continued T cell recruitment attenuated the antitumor effect of LSD1 depletion (Fig. 3d). Nevertheless, LSD1-deficient CD8^+^ TILs still showed a significantly higher cell number than wildtype CD8^+^ TILs in response to FTY720 treatment (Fig. 3c). These results suggest that, while continued CD8^+^ T cell recruitment is a critical basis for antitumor immunity, LSD1 depletion acts on the recruited CD8^+^ T cells to enhance their intratumoral expansion. Whether LSD1 depletion also promoted CD8^+^ T cell recruitment particularly at the later stage of tumor growth remained to be further addressed.

To further evaluate the expansion ability of tumor-resident CD8^+^ T cells with or without LSD1, we isolated CD8^+^ TILs from MC38 tumor-bearing *Cd4-Cre*^*+*^*Lsd1*^*f/f*^ (CD45.2^+^) and congenic wildtype (CD45.1^+^) mice and co-transferred those cells into TCRα KO recipient mice receiving MC38 tumors, followed by TIL analysis 14 days later (Fig. 3e). Among the transferred CD8^+^ TILs (TCRβ^+^), LSD1-deficient cells drastically exceeded wildtype cells in numbers, despite the fact that similar numbers of cells were co-transferred (Fig. 3f), again supporting that the intratumoral CD8^+^ T cells lacking LSD1 acquired better expansion capability.

### LSD1 depletion preserves the TCF1^+^PD-1^int^ progenitor subset of exhausted CD8^+^ T cells in the TME

**Fig. 4:**
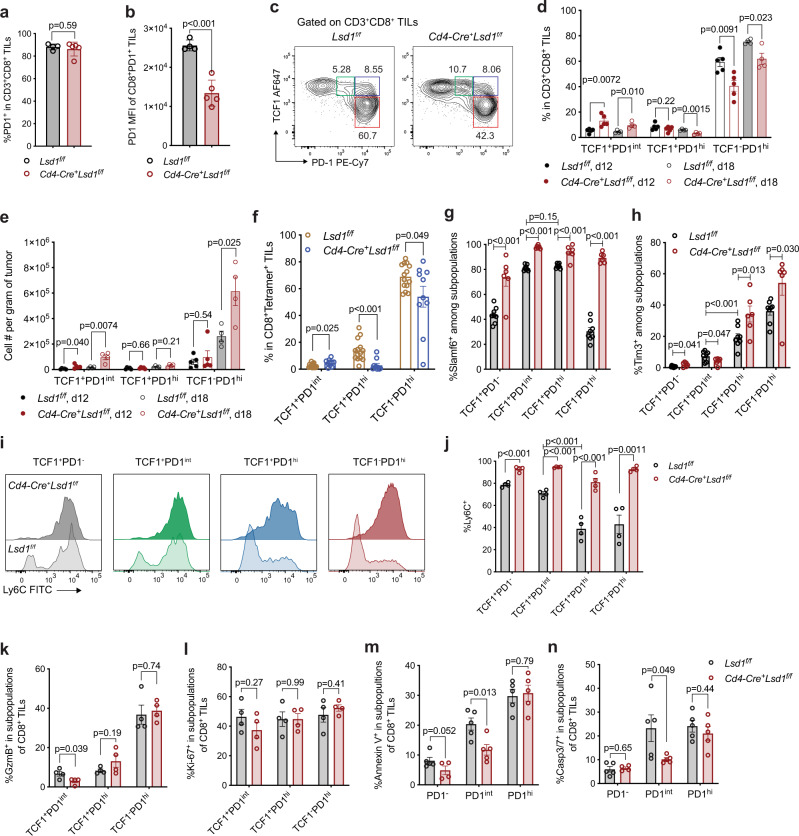
LSD1 depletion preserves intratumoral TCF1^+^PD-1^int^ progenitor exhausted CD8^+^ T cells. **a**, **b** Frequencies (**a**) and PD-1 MFI (**b**) of CD8^+^PD-1^+^ TILs on day 18 after MC38 tumor implantation (*Lsd1*^*f/f*^ group, *n* = 4; *Cd4-Cre*^*+*^*Lsd1*^*f/f*^ group, *n* = 5). **c**–**e** Representative flow plots (**c**), percentages (**d**) and cell numbers (**e**) of TCF1^+^PD-1^int^, TCF1^+^PD-1^hi^ and TCF1^-^PD-1^hi^ cells in CD8^+^ TILs analyzed by flow cytometry on day 12 and 18 after MC38 tumor implantation (*Lsd1*^*f/f*^ d12 group, *n* = 5; *Cd4-Cre*^*+*^*Lsd1*^*f/f*^ d12 group, *n* = 5; *Lsd1*^*f/f*^ d18 group, *n* = 4; *Cd4-Cre*^*+*^*Lsd1*^*f/f*^ d18 group, *n* = 4). **f** Percentages of TCF1^+^PD-1^int^, TCF1^+^PD-1^hi^ and TCF1^-^PD-1^hi^ cells in CD8^+^Tetramer^+^ TILs on day 18 after MC38-OVA tumor implantation (*Lsd1*^*f/f*^ group, *n* = 14; *Cd4-Cre*^*+*^*Lsd1*^*f/f*^ group, *n* = 10). **g**, **h**, Slamf6 (**g**) and Tim3 (**h**) expression by different subpopulations of CD8^+^ TILs analyzed by flow cytometry (*Lsd1*^*f/f*^ group, *n* = 8; *Cd4-Cre*^*+*^*Lsd1*^*f/f*^ group, *n* = 6). **i**, **j**, Representative flow plots of Ly6C expression (**i**) and percentages of Ly6C^+^ cells (**j**) in different subpopulations of CD8^+^ TILs (*n* = 4 per group). **k**, **l** GzmB (**k**) and Ki-67 (**l**) expression by different subpopulations of CD8^+^ TILs analyzed by flow cytometry on day 18 (*n* = 4 per group). **m**, **n** Cell apoptosis analysis by Annexin V staining (**m**) and active Casp3/7 staining (**n**) of CD8^+^ TILs isolated from MC38 tumors on day 14 (*n* = 5 per group). Data represent two independent experiments (**a**–**e**, **g**–**n**) or are pooled from three independent experiments (**f**) and are presented as mean ± SEM (**a**–**n**). Statistical significance was determined by two-sided unpaired t test (**a**–**n**). Source data are provided as a Source data file.

What is the biological basis that underlies the sustained expansion of LSD1-deficient CD8^+^ TILs? Recent studies have documented that among exhausted CD8^+^ T cells, the progenitor CD8^+^ T cells co-expressing PD-1 at an intermediate level (PD-1^int^) and TCF1 (encoded by *Tcf7* gene) essentially sustain intratumoral T cell expansion through self-renewing while continuously giving rise to numerically larger TCF1-negative, more differentiated cells^6,8^. In line with those reports, we showed that isolated CD8^+^ TILs with the PD-1^int^ phenotype, when adoptively transferred into MC38 tumor-bearing mice, retained a limited but detectable number of TCF1^+^PD-1^int^ cells in the TME, while converting into numerically more TCF1^-^PD-1^hi^ cells than did transferred PD-1^hi^ cells (Supplementary Fig. 4a–g). We thus set out to examine whether the progenitor exhausted CD8^+^ T cells were possibly regulated by LSD1. PD-1 expression, which is induced and sustained by chronic antigen stimulation^2^, was activated in both wildtype and LSD1-deficient CD8^+^ TILs (Fig. 4a). Although similar frequencies of PD-1^+^ cells were detected (Fig. 4a), the cell surface PD-1 protein in LSD1-deficient CD8^+^ TILs was expressed at a significantly lower level compared with that in wildtype CD8^+^ TILs of MC38 tumors (Fig. 4b). Among wildtype CD8^+^ TILs, TCF1^+^PD-1^int^ cells represented a subset much smaller than PD1^hi^ cells, which by and large lost TCF1 expression (TCF1^-^PD-1^hi^) (Fig. 4c–e). When LSD1 was depleted in T cells, we found the percentage of TCF1^+^PD-1^int^ cells significantly increased while the percentage of TCF1^-^PD-1^hi^ cells correspondingly decreased compared with their wildtype counterparts on both day 12 and 18 (Fig. 4d). Importantly, a larger cellular expansion of TCF1^+^PD-1^int^ TILs from day 12 to day 18 was observed when LSD1 was abrogated, which was associated with a greater expansion of the TCF1^-^PD-1^hi^ TILs, resulting in increased absolute cell numbers for both the TCF1^+^PD-1^int^ and TCF1^-^PD-1^hi^ subsets in the absence of LSD1 (Fig. 4e). In contrast, the TCF1^+^PD-1^hi^ subset was not elevated by LSD1 depletion (Fig. 4d, e), thus unlikely responsible for the increased expansion of LSD1-deficient CD8^+^ TILs, even though this subset might also contain the progenitor exhausted CD8^+^ T cells. Using MC38-OVA and tetramer staining, we further showed that tumor antigen-specific CD8^+^ TILs consistently displayed an elevated TCF1^+^PD-1^int^ subset in response to LSD1 loss (Fig. 4f), while in TdLNs, TCF1^+^PD-1^int^ subset remained numerically unaltered (Supplementary Fig. 5a–c). When T cell recruitment was blocked by FTY720 treatment starting on day 12 post tumor implantation, the TCF1^+^PD-1^int^ subset of LSD1-deficient CD8^+^ TILs appeared to be preferentially decreased to a level comparable to that of the WT counterpart in the next 10 days, while expectedly giving rise to numerically more TCF1^-^PD-1^hi^ cells than the WT counterpart (Supplementary Fig. 6a–c). Collectively, the TCF1^+^PD-1^int^ subset of exhausted CD8^+^ TILs seem to gain a stronger, but nonetheless limited, expansion ability upon LSD1 depletion, which, therefore, also underscores the need and importance of constant replenishment from TdLNs.

In addition to the MC38 tumor model, we found that LSD1 depletion also led to the expansion of TCF1^+^PD-1^int^ CD8^+^ TILs in pool size in the TRAMP-C2 tumor model, which was associated with the increased CD8^+^ T cell infiltration and the decreased PD-1 expression level by CD8^+^ TILs (Supplementary Fig. 7a–g). In B16/F10 tumor model, a substantial percentage (>30%) of CD8^+^ TILs showed the progenitor phenotype (PD-1^int^), which was further elevated close to 60% by LSD1 depletion (Supplementary Fig. 8a–c). Thus, a majority of LSD1-deficient CD8^+^ TILs retained the progenitor phenotype, which possibly compromises the timely conversion to more differentiated PD-1^hi^ cells that are responsible for generating cytotoxicity and immediate tumor control, contributing to an accelerated B16/F10 tumor growth. Indeed, the GzmB expression by CD8^+^ TILs was profoundly decreased by LSD1 depletion (Supplementary Fig. 8d, e). Introducing an OVA antigen to B16 tumors (B16-OVA) appeared to mitigate the defect in GzmB expression likely through promoting the conversion of PD-1^int^ cells to PD-1^hi^ cells (Supplementary Fig. 8f–j). Therefore, LSD1 depletion in T cells consistently elevates the progenitor subset of exhausted CD8^+^ T cell in the TME of a variety of tumor models, in spite of its differential impacts on tumor growth.

To ensure that the phenotype of CD8^+^ TILs was modulated by intrinsic LSD1 loss irrespective of CD4^+^ T cells, we used splenic CD8^+^ T cells from *Cd4-Cre*^*+*^*Lsd1*^*f/f*^ mice and littermate controls to reconstitute TCRα KO mice respectively, which were later implanted with MC38 tumors (Supplementary Fig. 9a). The analysis of CD8^+^ TILs on day 20 recapitulated the phenotype that LSD1-deficient CD8^+^ T cells were more expanded in the TME and displayed an elevated TCF1^+^PD-1^int^ subset (Supplementary Fig. 9b–d). Together, the above results demonstrate that LSD1 loss in CD8^+^ T cells intrinsically helps preserve the TCF1^+^PD-1^int^ progenitor exhausted cells.

We next examined the impact of LSD1 loss on different subsets of CD8^+^ TILs. Slamf6, recently reported as a marker of progenitor exhausted T cells^6^, was preferentially expressed by TCF1^+^PD-1^int^ and TCF1^+^PD-1^hi^ cells, and its expression was upregulated by LSD1 depletion across all subsets (Fig. 4g). A terminal exhaustion marker, Tim3, was significantly less expressed in TCF1^+^PD-1^int^ cells than TCF1^+^PD-1^hi^ cells (Fig. 4h), in line with the view that TCF1^+^PD-1^int^ cells represent the progenitor exhausted subset. Of note, LSD1 loss specifically downregulated Tim3 expression in the TCF1^+^PD-1^int^ subset but not in others (Fig. 4h). Additionally, in line with a recent report^35^, Ly6C expression was detected in a major part of the TCF1^+^PD-1^int^ subset, whereas only a minor part of the TCF1^+^PD-1^hi^ as well as TCF1^-^PD-1^hi^ subsets expressed Ly6C (Fig. 4i, j), indicating that Ly6C could be an additional marker to distinguish the TCF1^+^PD-1^int^ progenitor exhausted subset from others. Upon LSD1 loss, Ly6C expression was elevated in the TCF1^+^PD-1^int^ subset and sustained even in the TCF1^-^PD-1^hi^ subset (Fig. 4i, j). The TCF1^+^PD-1^int^ progenitor exhausted subset expressed much less GzmB than did the more differentiated TCF1^-^PD-1^hi^ subset in which GzmB expression was not interrupted by LSD1 loss (Fig. 4k). The expression of a proliferation marker Ki-67 remained largely unaltered by LSD1 depletion across all subsets (Fig. 4l). The unaltered proliferation rate of the TCF1^+^PD-1^int^ subset in response to LSD1 depletion suggests a potential survival advantage underlying the intratumoral persistence of these cells. Compared with CD8^+^PD-1^−^ TILs, antigen-experienced PD-1^int^ and PD-1^hi^ cells showed higher levels of apoptosis (Fig. 4m, n). Indeed, LSD1-deficient PD-1^int^ cells showed significantly less Annexin V staining and lower Caspase 3/7 activation than their wildtype counterparts (Fig. 4m, n). Importantly, such resistance was not observed in the PD-1^hi^ cells that mostly have lost TCF1 expression (Fig. 4m, n), indicating a unique mechanism of action of LSD1 that is restricted to the TCF1-expressing CD8^+^ TILs. Taken together, these results suggest that LSD1 depletion selectively improves the survival of the progenitor subset of exhausted CD8^+^ TILs contributing to the generation of an increasing number of more differentiated cells with better cytotoxicity.

### The LSD1/CoREST complex physically interacts with TCF1 and antagonizes its transcriptional activity

**Fig. 5:**
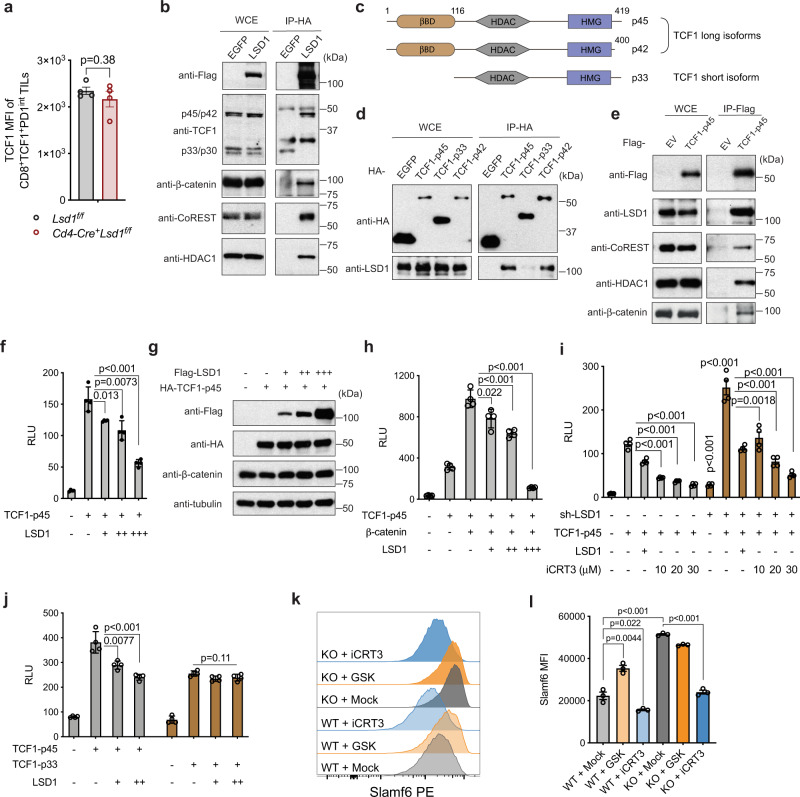
LSD1/CoREST complex physically interacts with TCF1 and antagonizes its transcriptional activity. **a** MFI of TCF1 in CD8^+^TCF1^+^PD-1^int^ TILs analyzed by flow cytometry (*n* = 4 per group). **b** The physical interaction between LSD1 and TCF1 isoforms as well as other proteins was examined by co-immunoprecipitation (co-IP) assays using whole-cell extract (WCE) of HEK293T cells transfected with FLAG-HA-tagged LSD1. **c** Schematic representation of different isoforms of mouse TCF1. **d** Co-IP assays precipitating HA-tagged TCF1 isoforms and detecting LSD1 in eluted IP fractions. **e** Co-IP assays precipitating Flag-tagged TCF1 (referring to p45 unless otherwise specified) and detecting components of the LSD1/CoREST complex in eluted IP fractions. **f**–**j** TCF/LEF reporter assay measuring relative luciferase units (RLU) in response to transfection with plasmids expressing indicated proteins (**f**–**j**, *n* = 4 per condition) or shRNA targeting LSD1 (**i**) and the treatment with iCRT3 (**i**) (*n* = 4 per condition). Protein expression for plasmid transfection in **f** is shown by immunoblotting (**g**). **k**, **l** Representative flow plots (**k**) and Slamf6 MFI (**l**) of splenic CD8^+^ T cells activated in vitro for 3 days with anti-CD3/anti-CD28 in the presence of vehicle, GSK2879552 or iCRT3 (*n* = 3 per treatment). Data represent two (**a**, **b**, **d**–**j**) or three (**k**, **l**) independent experiments and are presented as mean ± SEM (**a**) or mean ± SD (**f**, **h**–**j**, **l**). Statistical significance was determined by two-sided unpaired *t* test (**a**, **f**, **h**–**j**, **l**). Source data are provided as a Source data file.

TCF1 is a central transcription factor in maintaining the progenitor phenotype of the CD8^+^TCF1^+^PD-1^int^ T cells in tumors and chronic viral infections^6–8,11,13,36^. The observation that LSD1 acts selectively in the TCF1^+^PD-1^int^ cells prompted us to investigate possible interplays between LSD1 and TCF1. We first asked whether LSD1 regulated TCF1 expression and found that, in the CD8^+^TCF1^+^PD-1^int^ TILs, the protein level of TCF1 was not affected by LSD1 loss (Fig. 5a). We next asked whether LSD1 may impact TCF1 transcriptional activity through protein-protein interactions, as LSD1 is known to interact with a number of DNA-binding transcription factors^37^. Indeed, in HEK293T cells, antibodies against tagged LSD1 co-immunoprecipitated TCF1 protein, as well as CoREST and HDAC1, which are known components of the LSD1/CoREST complex (Fig. 5b)^38^. Interestingly, only the long isoforms of TCF1-p45/p42 with the β-catenin binding domain (βBD) was detected in the LSD1 immunoprecipitates, though both long (p45/p42) and short (p33 and p30) isoforms of TCF1 were expressed in these cells (Fig. 5b, c). In addition, β-catenin, which is a binding partner of the TCF1 long isoform important for TCF1-mediated transcriptional activation^39^, was also present in the LSD1 immunoprecipitates (Fig. 5b). To further confirm the selective interaction of LSD1 with the TCF1 long isoform, we expressed the epitope-tagged, TCF1 long isoform (p45 and p42) and short isoform (p33) for reciprocal co-immunoprecipitation (co-IP), respectively. We found that LSD1 protein was only present in the immunoprecipitates of the long isoform but not the short isoform of TCF1 (Fig. 5d), suggesting that the physical interaction of TCF1 with LSD1 involves the βBD domain present only in the TCF1 long isoform. However, whether LSD1 or other components of the LSD1/CoREST complex directly bind to the βBD domain of TCF1 long isoform or indirectly through its binding to β-catenin or other TCF1-interacting proteins remains to be further investigated. As expected, several other components of the LSD1/CoREST complex were also pulled down by the TCF1 long isoform in the co-IP assays (Fig. 5e). Thus, these results suggest that the LSD1/CoREST complex physically interacts with the long isoform of TCF1.

LSD1 represses gene expression by catalyzing the removal of the methyl groups from histone H3K4me1/2^40^. The fact that LSD1 interacts with TCF1 raised the possibility that LSD1 may compromise TCF1-mediated transcriptional activation. To test this possibility, we used a well-established reporter assay designed to monitor the transcriptional activation mediated by TCF/LEF family members^41^. Indeed, the transcriptional activity of the TCF1 long isoform was suppressed by the co-transfected LSD1 in a dose-dependent manner without altering protein expression of the ectopic TCF1 or endogenous β-catenin (Fig. 5f, g). Co-transfection of β-catenin strongly elevated the transcriptional activity of the TCF1 long isoform, which could also be largely attenuated by overexpressing LSD1 (Fig. 5h). Conversely, LSD1 downregulation significantly increased the transcriptional activity mediated by the endogenous TCF/LEF as well as the ectopically expressed TCF1 long isoform (Fig. 5i). β-catenin is a transcriptional co-activator of TCF1 and the physical interaction of β-catenin with TCF1 is important for transcriptional regulation, as disruption of their interaction by the inhibitor, iCRT3^42^, severely dampened the transcriptional activation mediated by TCF1/β-catenin (Fig. 5i). Our findings suggest that LSD1 compromises TCF1-mediated transcription by physically interacting with TCF1. Consistent with this hypothesis, the transcriptional activity of the TCF1 short isoform, which does not interact with LSD1, was not affected by changes of LSD1 protein levels (Fig. 5j). Taken together, these data suggest that the physical interaction of LSD1 with TCF1 suppresses its transcriptional activity, although detailed molecular mechanisms remain to be elucidated.

To determine the biological significance of this interplay in T cells, we activated CD8^+^ T cells isolated from mouse spleens by polyclonal stimulation and examined the transcriptional activity of the endogenous TCF1 by measuring the expression of its target gene *Slamf6*^6,11^. As a control. disrupting TCF1/β-catenin complex by iCRT3 compromised Slamf6 induction in activated CD8^+^ T cells as expected (Fig. 5k, l). In contrast, inhibition of LSD1 by GSK2879552 during T cell activation significantly upregulated Slamf6 expression, which was recapitulated by LSD1 genetic ablation (Fig. 5k, l). Thus, LSD1 antagonizes the transcriptional activity of TCF1 in CD8^+^ T cells.

### LSD1 inhibition unleashes TCF1-controlled gene expression involved in intratumoral T cell persistence

**Fig. 6:**
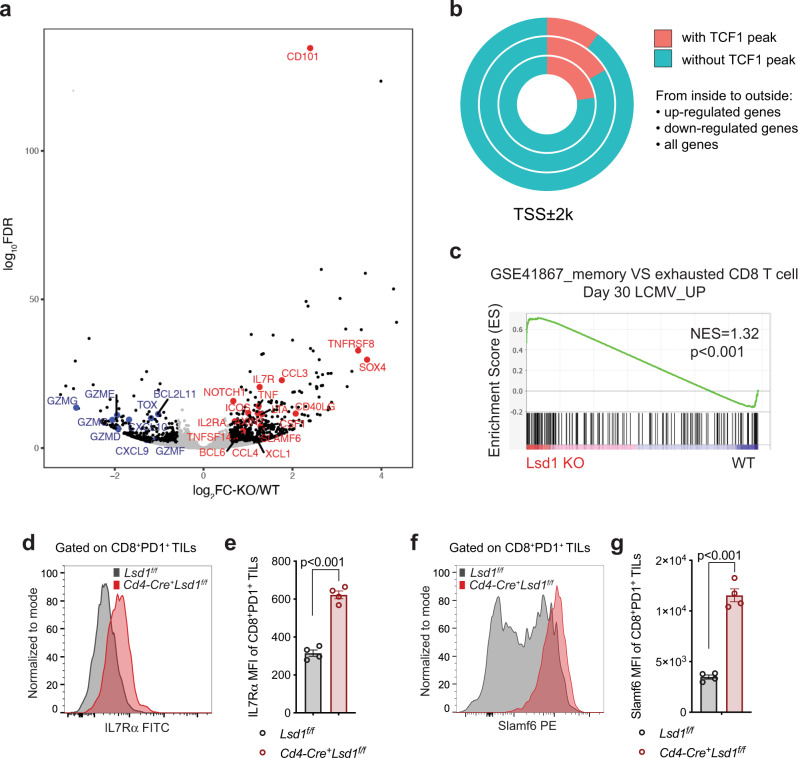
LSD1 loss unleashes TCF1-controlled gene expression in CD8^+^ TILs. **a** A volcano plot showing differential gene expression by LSD1-deficient versus wildtype CD8^+^CD44^+^PD-1^+^ TILs of MC38 tumors (*n* = 4 per group, FC > 1.5 and FDR < 0.01 as the cutoff). Non-gray colored dots mean significant difference. **b** Percentages of genes bound by TCF1 among the differentially expressed genes in LSD1-deficient versus wildtype CD8^+^ TILs or among total genes in genome. **c** GSEA analysis in *Lsd1* KO versus WT CD8^+^ TILs (*n* = 4 per group). Gene list was ranked with signed (from log_2_FC) likelihood ratio from *Lsd1* KO versus WT comparison. Kolmogorov–Smirnov test was used to determine the enrichment score. **d**, **e** A representative flow plot of IL7Rα expression (**d**) and IL7Rα MFI (**e**) of MC38 tumor-infiltrating CD8^+^PD-1^+^ T cells (*n* = 4 per group). **f**, **g** A representative flow plot of Slamf6 expression (**f**) and Slamf6 MFI (**g**) of MC38 tumor-infiltrating CD8^+^PD-1^+^ T cells (*n* = 4 per group). Data represent two independent experiments (**d**–**g**) and are presented as mean ± SEM (**e**, **g**). Statistical significance was determined by two-sided unpaired *t* test (**e**, **g**). Source data are provided as a Source data file.

On the basis of the above data, we hypothesized that LSD1 loss augmented the transcriptional program mediated by TCF1 that maintained a progenitor subset of intratumoral CD8^+^ T cells for extended tumor control. To address this possibility, we isolated MC38 tumor-infiltrating CD8^+^CD44^+^PD1^+^ T cells for transcriptomic analysis by RNA-seq (Supplementary Fig. 10a, b). We found 561 genes upregulated and 351 genes downregulated (fold change > 1.5 and FDR < 0.01) in LSD1-deficient versus wildtype cells (Fig. 6a and Supplementary Data 1), and the differentially expressed genes were enriched in immune-related pathways (Supplementary Fig. 10c). Of note, 22.8% of the upregulated genes were TCF1 direct targets in our analysis of a previously reported ChIP-seq dataset^43^, in comparison to 10.0% of genome bound by TCF1 (Fig. 6b)^43^, reflecting an enrichment of TCF1-modulated genes among the upregulated genes. Gene-set enrichment analysis (GSEA) revealed that a memory CD8^+^ T cell signature was enriched in the LSD1-deficient CD8^+^ TILs (Fig. 6c). A number of genes, for instance *Il7r*, *Slamf6*, and *Bcl6*, which are TCF1 targets and associated with the progenitor phenotype of CD8^+^ TILs^6,43^, were significantly upregulated by LSD1 depletion (Fig. 6a). Interestingly, the expression of a few cytokines, including TNFα, LTA, XCL1, CCL3, and CCL4, was also induced in the LSD1-deficient CD8^+^ TILs, while several murine-specific granzymes (granzyme C, D, E, F, and G) of unknown activity were decreased (Fig. 6a)^44^. Consistent with the resistance to apoptosis (Fig. 4m, n), *Bcl2l11* encoding pro-apoptotic BIM was reduced in the LSD1-deficient CD8^+^ TILs (Fig. 6a).

To further confirm the RNA-seq data, we analyzed gene expression by flow cytometry in the MC38 tumor model. Indeed, intratumoral CD8^+^ T cells without LSD1 showed increased IL7Rα and Slamf6 (Fig. 6d–g). These phenotypes were consistently recapitulated in the TRAMP-C2 tumor model (Supplementary Fig. 11a–d). In line with the progenitor phenotype of CD8^+^PD-1^int^ T cells, this population expressed higher levels of IL7Rα and Slamf6 than did the CD8^+^PD-1^hi^ T cells (Supplementary Fig. 11e–j). Notably, LSD1 loss significantly upregulated IL7Rα and Slamf6 in the CD8^+^PD-1^int^ T cells, in contrast to a mild effect in the CD8^+^PD-1^hi^ T cells (Supplementary Fig. 11e–j), further supporting the functional significance of the proposed interplay between LSD1 and TCF1 in the progenitor exhausted CD8^+^ TILs. In line with the decreased TOX expression observed by RNA-seq analysis (Fig. 6a), LSD1 depletion compromised the differentiation of TCF1^+^ cells into a more exhausted state marked by TOX expression (Supplementary Fig. 11k–m)^23–25^. The gene expression data thus suggest that TCF1-controlled transcription network involved in CD8^+^ T cell survival and self-renewal is augmented in response to LSD1 depletion.

### Targeting LSD1 promotes long-lasting responses to anti-PD1 treatment

**Fig. 7:**
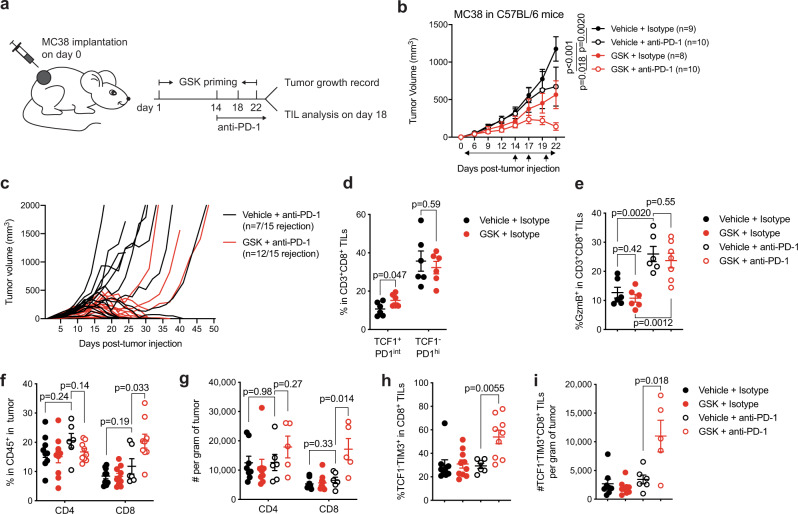
Targeting LSD1 enhances long-lasting response to anti-PD-1 treatment. **a** Experimental design of administering GSK2879552 and PD-1 blocking antibodies. **b** Tumor growth curves of wildtype mice inoculated with MC38 tumor cells and treated with GSK2879552 or vehicle control in combination with anti-PD-1 or isotype control. **c** Individual tumor growth curves over time in response to anti-PD1 treatment alone or in combination with GSK2879552. **d**, **e** Percentages of TCF1^+^PD-1^int^ and TCF1^-^PD-1^hi^ cells (**d**) or GzmB^+^ cells (**e**) in CD8^+^ TILs (Vehicle + Isotype, *n* = 6; GSK + Isotype, *n* = 6; Vehicle + anti-PD-1, *n* = 6; GSK + anti-PD-1, *n* = 7). **f**, **g**, Percentages (**f**, *n* = 9, 10, 6, 9, respectively) and cell numbers (**g**, *n* = 8, 9, 6, 5, respectively) of tumor-infiltrating CD3^+^CD4^+^ T cells and CD3^+^CD8^+^ T cells in gated CD45^+^ leukocytes. **h**, **i** Frequencies (**h**, *n* = 9, 10, 6, 9, respectively) and cell numbers (**i**, *n* = 8, 9, 6, 5, respectively) of CD8^+^TCF1^-^TIM-3^+^ TILs. Data represent two independent experiments (**b**, **d**–**i**) or are pooled from two independent experiments (**c**), and are presented as mean ± SEM (**b**, **d**–**i**). Sample sizes are as indicated. Statistical significance was determined by two-way ANOVA (**b**) or two-sided unpaired *t* test (**d**–**i**). Source data are provided as a Source data file.

Among exhausted CD8^+^ T cells, the intratumoral TCF1^+^PD-1^int^ progenitor subset has been reported to be the prominent determinant of effective responses to PD-1 blockade^6,8,9,13^. The remarkable effect of LSD1 depletion on sustaining the progenitor exhausted CD8^+^ TILs prompted us to examine whether targeting LSD1 could improve tumor responses to anti-PD-1 treatment. Since tumor antigen-driven T cell exhaustion has been suggested to occur early in tumor development^45^, we initiated GSK2879552 treatment on the second day after tumor implantation, followed by anti-PD1 treatment two weeks later when tumors were well-established (Fig. 7a). The growth of MC38 tumors was significantly restrained by either GSK2879552 or anti-PD-1 treatment, and the combination of these two treatments displayed a cooperative effect on controlling tumor growth (Fig. 7b). Notably, tumors primed with GSK2879552 treatment mostly showed prolonged responses to PD-1 blockade with a significant increase of tumor rejection rate (80%, 12/15 versus 47%, 7/15) (Fig. 7c). Thus, systemic treatment with an LSD1 inhibitor enables long-lasting responses to PD-1 blockade.

To elucidate the mechanisms underlying the long-lasting response of GSK2879552-primed tumors to PD-1 blockade, we conducted comparative analyses of tumor immune microenvironment in response to different treatment. GSK2879552 and/or anti-PD1 treatment had no overt effect on the infiltration of MDSCs, DCs or NK cells, but GSK2879552 exerted a suppressive effect on GzmB expression by NK cells (Supplementary Fig. 12a–d). Similar to the results from the genetic study (Figs. 2h and 4e), GSK2879552 significantly increased the pool size of the TCF1^+^PD-1^int^ progenitor exhausted CD8^+^ TILs (Fig. 7d), but did not affect the overall GzmB expression by CD8^+^ TILs (Fig. 7e). Upon anti-PD-1 treatment, GzmB expression by CD8^+^ TILs was strongly increased independent of prior GSK2879552 treatment (Fig. 7e). In fact, the frequency of GzmB-expressing CD8^+^ TILs was comparable in tumors treated with anti-PD-1 alone or together with GSK2879552 (Fig. 7e). Under this treatment regimen (Fig. 7a), anti-PD-1 treatment alone did not have an overt effect on the infiltration of CD4^+^ or CD8^+^ TILs, whereas it significantly and preferentially expanded intratumoral CD8^+^ T cells only when tumors were primed with GSK2879552 treatment (Fig. 7f, g and Supplementary Fig. 12e). In addition, GSK2879552 treatment increased the generation of the TCF1^−^TIM-3^+^ terminally exhausted CD8^+^ TILs upon PD-1 blockade (Fig. 7h, i), likely through promoting their conversion from a larger pool of the TCF1^+^PD-1^int^ progenitor exhausted CD8^+^ TILs (Fig. 7d)^6,8,9,11^. Taken together, these data suggest that LSD1 inhibition preserves the progenitor exhausted CD8^+^ TILs and sustains intratumoral T cell expansion, resulting in long-lasting responses of tumors to anti-PD-1 treatment.

## Discussion

The ineffective reinvigoration of exhausted T cells accounts for the lack of durable responses to PD-1 blockade therapy. Among heterogeneous and exhausted antigen-specific CD8^+^ T cells in chronic viral infections and cancer, the TCF1^+^ progenitor cells preferentially respond to PD-1 blockade^6–9,11^. Nevertheless, proliferation of the progenitor cells driven by antigen stimulation progressively induces their conversion to terminally differentiated cells, even when PD-1 pathway is blocked. Although not formally proven experimentally, this represents a plausible reason for the transient response to anti-PD-1 treatment^2^. Thus, maintaining and/or expanding such progenitor CD8^+^ T cell population in the TME may lead to a long-lasting tumor response to anti-PD-1 treatment. Both our genetic and chemical biology approaches support the notion that LSD1 enforces an epigenetic program, which restrains the progenitor pool and promotes T cell terminal exhaustion in the TME, possibly through antagonizing TCF1-mediated transcription. Therefore, manipulating LSD1 in T cells results in a long-lasting response of tumors to anti-PD1 therapy.

Epigenetic regulation is critically involved in almost all aspects of T cell biology including lineage commitment, development, activation, differentiation, and memory formation^46^. The role of LSD1 in those biological processes remains largely unexplored. We used CD4-Cre in order to efficiently delete floxed *Lsd1* alleles in TCRαβ^+^ T cells, and a noticeable loss of LSD1 protein was observed as early as the CD4 or CD8 single positive (SP) stage, although the CD4-driven Cre recombinase is expected to be expressed earlier at the CD4/CD8 double positive (DP) stage^47^. Since LSD1 protein in the DP thymocytes of *Cd4-Cre*^*+*^*Lsd1*^*f/f*^ mice was detected at a similar level as the wildtype counterpart, these data suggest that key events specific to DP thymocytes, such as TCRα rearrangements and positive selection, are unlikely affected in *Cd4-Cre*^*+*^*Lsd1*^*f/f*^ mice. Consistently, DP thymocytes remain mostly unaltered upon *Lsd1* deletion. These observations suggest that the improved antitumor immunity in *Cd4-Cre*^*+*^*Lsd1*^*f/f*^ mice is likely due to a role of LSD1 in regulating the activation and differentiation of mature T cells upon tumor antigen stimulation, which is further supported by our chemical approach with the LSD1 inhibitors.

Persistent antigen stimulation is the key factor driving CD8^+^ T cell exhaustion. In the immunogenic MC38 tumor model, the pool size of the progenitor CD8^+^ TILs was small (<8%), so the long-term antitumor immunity was likely limited by the maintenance of the progenitor subset of exhausted CD8^+^ TILs, which can be significantly improved by perturbing T cell-intrinsic LSD1. In contrast, in poorly immunogenic B16 melanoma model, a substantial portion (>30%) of CD8^+^ TILs showed the progenitor phenotype, which was further elevated close to ~60% by the LSD1 depletion. Thus, a majority of LSD1-deficient CD8^+^ TILs retained the progenitor phenotype, thereby compromising the timely conversion to terminally exhausted CD8^+^ TILs that are responsible for the immediate tumor-killing effect and short-term tumor control. As a result, B16 tumors displayed an accelerated outgrowth shortly after implantation in T cell-specific LSD1 knockout mice. Hence, perturbation of T cell-intrinsic LSD1 consistently increases the progenitor subset of exhausted CD8^+^ T cell in the TME in various tumor models, but drives tumor outgrowth differently depending on tumor context. The impact of targeting T cell-intrinsic LSD1 on B16 tumor growth is opposite to our previous finding that the ablation of tumor cell-intrinsic LSD1 enhances tumor immunogenicity, suppresses tumor growth and overcomes tumor primary resistance to PD1 blockade therapy^31^. This highlights the importance of tumor stratification as well as targeted drug delivery approach for reaching a combinatory antitumor effect of LSD1 inhibitors with PD1 blocking antibodies.

T cell activation, differentiation, and exhaustion, driven by tumor antigens-induced chronic stimulation and regulated by tumor microenvironmental factors, display significant remodeling of the chromatin landscapes^6,16,18^. The importance of chromatin regulation in such biological processes has been well recognized, but how it actually works to influence T cell activation and differentiation remains incompletely understood. Our study uncovers a critical role of the histone demethylase LSD1 in controlling the balance between the progenitor exhausted and the terminally exhausted CD8^+^ TILs. The observation that LSD1 only interacted with the long but not short isoforms of TCF1 highlights an important role of LSD1 in the progenitor exhausted CD8^+^ TILs, since the long isoform of TCF1 has been suggested to sustain the stem-like properties^8,48,49^, which is likely to be suppressed by LSD1 through their interaction. Once the progenitor exhausted CD8^+^ TILs lose the expression of TCF1 long isoform and subsequently convert to terminally exhausted cells, LSD1 may no longer have substantial effects. Indeed, we found apoptosis of the terminally exhausted cells largely unaffected by LSD1 loss, in contrast to the reduced apoptosis in the progenitor exhausted cells. The presence of β-catenin binding domain in the long isoform of TCF1 could mediate the recruitment of β-catenin to the interacting protein complex, which was detected in the TCF1 p45 as well as LSD1 immunoprecipitates. However, it remains to be determined whether the interaction with β-catenin is necessary for the function of the long isoform of TCF1, particularly given a long-standing debate on the necessity of β-catenin in memory T cell formation^50–52^. Alternatively, other co-factors, such as ATF2^53^, may also bind to and regulate the transcriptional activity of the long isoform of TCF1 independent of the canonical Wnt-β-catenin signaling.

The physical interaction of LSD1/CoREST complex with TCF1 is consistent with the idea that LSD1 needs other components of this complex to mediate gene repression^38,54,55^, and supports the involvement of other components of the complex in regulating CD8^+^ T cell exhaustion. Since LSD1 also mediates demethylation of non-histone proteins^37^, whether LSD1 influences the function of TCF1 via demethylation of TCF1 or its binding partners remains to be determined. Collectively, understanding the precise biochemical mechanism of how LSD1 interacts with TCF1 to impact its transcription program requires further investigations in the progenitor exhausted CD8^+^ T cells. Additionally, future investigations of histone modifications, including methylation and acetylation, in different subsets of CD8^+^ TILs will provide more in-depth insights into the chromatin mechanisms underlying the maintenance and differentiation of the progenitor CD8^+^ TILs.

LSD1 is upregulated in a variety of cancers and mostly plays a tumor-promoting role through multiple mechanisms including maintaining the stemness of tumor cells^56–58^, supporting tumor cell proliferation^30,59^, and suppressing antitumor immunity^31^. Thus, LSD1 has been actively explored as a target for cancer treatment. Notably, LSD1 also plays critical physiological roles in haematopoiesis^60^ as well as neuronal differentiation and function^61–63^, and the administration of some irreversible LSD1 inhibitors has been found to cause adverse toxicities in some cancer clinical trials^64^. In contrast, some other irreversible LSD1 inhibitors and also recently developed reversible LSD1 inhibitors tested appear to have no dose-limiting toxicities in recent clinical studies^64^. The crucial role of LSD1 in CD8^+^ T exhaustion that we identify and report here warrants further consideration of LSD1 inhibition in combination with PD-1 pathway inhibitors in cancer therapy. In one approach, current LSD1 inhibitors with tolerable toxicities could be explored to prime CD8^+^ T cells for a short duration to improve their sustainable responses to PD-1 blockade. In another approach, based on the unique mechanism of action of LSD1 in intratumoral CD8^+^ T cells mediated by its interaction with TCF1 long isoform, we propose that antagonists, either small molecules or biologics that disrupt the interaction between LSD1 and the long isoform TCF1, could be developed in cancer checkpoint blockade therapy. Moreover, both approaches of inhibiting LSD1 or genetic ablation of LSD1 could also be exploited to enhance the persistence of cytotoxic T cells in adoptive T-cell therapy for cancer. Further studies are warranted to stratify tumors that may benefit from the combination of LSD1 perturbation with immunotherapy.

In summary, our study uncovers an important role of LSD1 and its mechanism of action in regulating intratumoral CD8^+^ T cells and its impact on tumor growth control. Our study also highlights the translational significance of combining LSD1 inhibitors with PD-1 blockade in cancer treatment, in which LSD1 inhibition in CD8^+^ T cells potentiates the progenitor phenotype and thus promotes long-lasting responses to PD-1 blockade. Our current data suggest that certain tumors respond to the combination of LSD1 inhibition and PD-1 blockade, thus identification of biomarkers is necessary to help stratify patients in order to significantly enhance the clinical development and benefits of this approach. The identification of a specific mechanism of action of LSD1 in intratumoral CD8^+^ T cells also points to a promising avenue for targeting LSD1 in cancer immunotherapy through specifically disrupting the physical interaction between LSD1 and the long isoform of TCF1, a pivotal regulator driving the progenitor phenotype maintenance.

## Methods

### Cell culture

MC38, B16, and HEK293T cells were cultured in DMEM medium containing 10% heat-inactivated FBS and 1% penicillin/streptomycin in a 5% CO_2_ incubator at 37 °C. TRAMP-C2 cells were cultured in DMEM medium supplemented with 5% Nu-Serum IV, 5% heat-inactivated FBS, 5 μg/ml bovine insulin, 10 nM DHEA and 1% penicillin/streptomycin in a 5% CO_2_ incubator at 37 °C. Isolated CD8^+^ T cells were cultured in R10 medium (RPMI 1640 supplemented with 10% FBS, 1% penicillin/ streptomycin, 12 mM HEPES and 50 μM 2-mercaptoethanol). MC38 cell line was a gift from Dr. Arlene Sharpe and B16/F10 was a gift from Dr. David Fisher. Both cell lines were originally purchased from ATCC. MC38-OVA cell line was a gift from Dr. Ana Anderson and B16-OVA cell line was gift from Dr. Nick Haining. HEK293T and TRAMP-C2 cell line were purchased from ATCC.

### Mice

Six to ten-week-old mice were used for all experiments. Wildtype C57BL/6 mice were purchased from The Jackson Laboratory. *Cd4-Cre* transgenic mice (purchased from The Jackson Laboratory, stock #017336) were crossed with *Lsd1* floxed mice (gifts from Dr. Stuart Orkin at Boston Children’s Hospital) to generate *Cd4-Cre*^*+*^*Lsd1*^*f/f*^ knockout mice and *Lsd1*^*f/f*^ littermate control mice. Immunodeficient TCRα knockout mice were originally purchased from The Jackson Laboratory (stock #002116) and bred in-house. CD45.1 mice were purchased from The Jackson Laboratory (stock #002014). Prior to all experiments, purchased mice were allowed to acclimate to housing conditions at the Boston Children’s Hospital Animal Facility for one week. Mice were subcutaneously inoculated with tumor cells and were euthanized when tumor volumes exceed 2000 cubic millimeters. All experimental mice were housed in specific pathogen-free conditions and all animal procedures were performed in accordance with animal care guidelines and with the prior approval by the Boston Children’s Hospital Institutional Animal Care and Use Committee. Male animals were used for TRAMP-C2 related experiments. Female animals were mostly used for other experiments.

### Mouse subcutaneous tumor models

2.5 × 10^5^ or 5 × 10^5^ MC38 or B16, or 10^6^ TRAMP-C2 cells in 1× PBS were subcutaneously injected into the right hind flank of each pre-shaved wildtype, *Cd4-Cre*^*+*^*Lsd1*^*f/f*^ or *Lsd1*^*f/f*^ mouse to establish tumors. Both male and female mice were used in MC38 and B16 tumor models and only male mice were used in TRAMP-C2 tumor model. Tumors were manually measured and recorded using a digital caliper starting from day 6 every 2–3 days. Tumor size was calculated using the following formula: ½ × length × width^2^. Mice reached the endpoint when tumor volumes were over 2000 mm^3^.

For T cell reconstitution, CD8^+^ T cells were isolated from spleens of *Cd4-Cre*^*+*^*Lsd1*^*f/f*^ or *Lsd1*^*f/f*^ mice by magnetic separation using CD8a (Ly-2) microbeads (Miltenyi Biotec, cat#130-117-044) according to the instruction manual. The isolated CD8^+^ T cells were resuspended in 1× PBS and intravenously transferred into TCRα knockout mice at 8 million cells per mouse. On the next day, reconstituted mice were inoculated with 2.5 × 10^5^ MC38 cells for tumor growth and TIL analysis experiments.

For drug treatments, mice were administered with rat IgG2a isotype control (BioXCell, clone 2A3), anti-PD-1 (BioXCell, clone 29F.1A12), vehicle or GSK2879552 (MedChem Express, cat#HY-18632) at the time points shown in the figures. 100 μg anti-PD-1 or isotype control were injected intraperitoneally into each mouse every 3 days as indicated in the figures. GSK2879552 or vehicle was injected intraperitoneally into each mouse every day after tumor inoculation at a dose of ~1.5 mg/kg body weight. Prior to treatments, mice were randomized such that treatment groups had similar average tumor volumes prior to treatment initiation. FTY720 treatment was initiated at day 12 post-tumor inoculation at a dose of 1 mg/kg body weight and followed by intraperitoneal injection at a dose of 0.2 mg/kg daily until tumor collection.

### Isolation and in vitro activation of splenic CD8^+^ T cells

Spleens were harvested from *Cd4-Cre*^*+*^*Lsd1*^*f/f*^ and *Lsd1*^*f/f*^ mice and CD8^+^ T cells were isolated by magnetic separation using CD8a (Ly-2) microbeads (Miltenyi Biotec, cat#130-117-044) according to the instruction manual. The isolated CD8^+^ T cells were resuspended in R10 medium at 5 × 10^5^/ml and plated onto 24-well plates pre-coated with 3 μg/ml anti-CD3 (BioLegend, cat#100340), supplemented with 2 μg/ml anti-CD28 (BioLegend, cat#102116) and 25 ng/ml IL-2 (PeproTech, cat#200-02). CD8^+^ T cells were treated with GSK2879552, iCRT3 (MedChem Express, cat#HY-103705) or vehicle as indicated. After 72 h of in vitro stimulation, activated CD8^+^ T cells were collected, stained with antibodies against surface markers, and when needed, fixed and permeabilized with a Foxp3/Transcription Factor Staining Buffer Set (eBioscience, cat#00-5523-00), followed by intracellular staining. 7-AAD Viability Staining Solution (BioLegend, cat#420404) was added lastly to exclude dead cells for unfixed cells, and LIVE/DEAD™ Fixable Near-IR Dead Cell Stain Kit (Thermo Fisher Scientific, cat#L10119) was used for cells subjected to fixation. Flow cytometry data were acquired on a BD LSR II or BD FACSymphony using FACSDiva v8.0.1 and analyzed by FlowJo 10.4 software.

### Tumor-infiltrating leukocyte analysis by flow cytometry

5 × 10^5^ MC38 or B16, or 10^6^ TRAMP-C2 cells were subcutaneously injected into the right flank of individual mouse and tumors were harvested on day 12–20 as indicated after tumor inoculation. Tumors were minced into small pieces and digested in RPMI1640 medium containing 400 U/ml type I collagenase (Worthington Biochemical Corporation, cat#LS004194) and 100 μg/ml DNase I (Sigma-Aldrich, cat#10104159001) for 20–30 min at 37 °C. Digested tumor tissue samples were neutralized with R10 medium and then filtered through a 70 μM cell strainer to obtain single cell suspensions. Samples were pelleted and resuspended in 5 ml of 40% Percoll (GE Healthcare, cat#17-0891-01) and underlayed by 3 ml of 70% Percoll in a 15 ml conical tube. After centrifugation at 800 g for 20 min with break set at 1, leukocytes were enriched at the interface between 40 and 70% Percoll gradient. Collected leukocytes from the gradient interface were then resuspended in ACK lysis buffer to remove red blood cells and then stained with antibodies against surface markers and intracellular proteins, or SIINFEKL H-2Kb Tetramer (NIH Tetramer Core Facility) as needed. For cytokine staining, leukocytes were first stimulated with 1 μM OVA_257–264_ peptides (Anaspec, cat#AS-60193-1) or PMA/Ionomycin in the presence of Golgiplug for 4 h. Stained tumor-infiltrating leukocyte (TIL) samples were run on a BD LSR II or BD FACSymphony using FACSDiva v8.0.1 and analyzed by FlowJo software. All antibodies were purchased from BioLegend, Thermo Fisher Scientific or BD Biosciences and were used at 1:200 dilution: SIINFEKL H-2K^b^ Tetramer (NIH Tetramer Core Facility); LIVE/DEAD™ Fixable Near-IR Dead Cell Stain Kit (ThermoFisher Scientific, L10119); IgG2a isotype control (clone 2A3, BioXCell, BE0089); anti-PD-1 (BioXCell, clone 29F.1A12, BE0273); CD45.2, BV421 (clone 104, BioLegend, Cat#109831); CD45.2, PE (clone 104, BioLegend, Cat#109807); CD45.1, FITC (clone A20, BioLegend, Cat#110705); CD3e, BV510 (clone145-2C11, BioLegend, Cat#100353); TCRb, BV510 (clone H57-587, BioLegend, Cat#109233); CD4, APC (clone RM4-5, BioLegend, Cat#100516); CD8b, APC/Cy7 (clone YTS156.7.7, BioLegend, Cat#126619); CD8b, PE (clone YTS156.7.7, BioLegend, Cat#126607); CD8a, BV605 (clone 53-6.7, BioLegend, Cat#100743); CD8a, BV510 (clone 53-6.7, BioLegend, Cat#100751); Foxp3, PE (clone FJK-16s, ThermoFisher Scientific, Cat#12-5773-82); Granzyme-B, FITC (clone GB11, BioLegend, Cat#515403); Ki-67, PercCP-Cy5.5 (clone B56, BD Biosciences, Cat#561284); CD44, FITC (clone IM7, BioLegend, Cat#103005); CD62L, BV510 (clone MEL-14, BioLegend, Cat#104441); CD62L, PE (clone MEL-14, BioLegend, Cat#104407); CD16/32 (clone 93, BioLegend, Cat#101320); 7-AAD viability staining solution (BioLegend, Cat#420404); CD11b, BV605 (clone M1/70, BioLegend, Cat#101237); Gr-1, APC-Cy7 (clone RB6-8C5, BioLegend, Cat#108423); TNFa, FITC (cloneMP6-XT22, BioLegend, Cat#506303); IL-2, PerCP-Cy5.5 (cloneJES6-5H4, BioLegend, Cat#503821); IFN-g, PE (clone XMG1.2, BioLegend, Cat#104407); PD-1, PE-Cy7 (29F.1A12, BioLegend, Cat#135215); Tim-3, PE(RMT3-23, BioLegend, Cat#119703); Tim-3, APC (RMT3-23, BioLegend, Cat#119705); TCF1/TCF7, AF647 (C63D9, CST, Cat#6709S); CD127, PE-Cy7 (A7R34, BioLegend, Cat#135013); CD127, FITC (A7R34, BioLegend, Cat#135007); TOX, PE (TXRX10, eBiosciences, Cat#12-6502-80); Slamf6, PE (clone 13G3, eBiosciences, Cat#12-1508-80).

### CD8^+^ TIL co-transfer assay

Congenic CD45.1^+^ and *Cd4-Cre*^*+*^*Lsd1*^*f/f*^ (CD45.2^+^) mice were implanted with 5 × 10^5^ MC38 tumor cells for 14 days, followed by tumor excision and TIL isolation as described above. CD8^+^ TILs were further isolated by magnetic separation using CD8a (Ly-2) microbeads. The number of CD8^+^ TILs was quantified by staining a small aliquot with antibodies against CD45, CD3, and CD8 for flow cytometry. CD45.1^+^CD8^+^ wildtype TILs and CD45.2^+^CD8^+^ LSD1-deficient TILs were then mixed in equal numbers and intravenously transferred into TCRα knockout recipient mice at 20,000 cells per mouse. On the next day, recipient mice were implanted with 5 × 10^5^ MC38 tumor cells. After 14 days, TILs were isolated as described above and the frequencies of CD45.1^+^ and CD45.2^+^ cells among TCRβ^+^CD8^+^ TILs were analyzed by flow cytometry.

### In vivo transfer and persistence assay

7-week-old female C57BL/6J mice were subcutaneously implanted with 5 × 10^5^ MC38 cells on day 0. CD8^+^ TILs were isolated from MC38 tumors on day 20 as described above and stained with antibodies against CD45.2 (clone 104), CD3 (clone145-2C11), CD8 (clone YTS156.7.7), PD-1 (clone 29F.1A12) and TIM-3 (clone RMT3-23). 7-AAD Viability Staining Solution was used to exclude dead cells. PD-1^int^Tim-3^-^ or PD-1^hi^Tim-3^-^ CD8^+^ TILs were sorted and resuspended in PBS. 10,000 sorted cells of these two populations were transferred via intravenous tail vein injection into TCRα KO mice respectively, which were implanted with 2.5 × 10^5^ MC38 cells on the next day. TILs were isolated after 12–14 days of tumor implantation and analyzed to determine the number and immunological phenotype of the transferred CD8^+^ cells.

### Apoptosis assay

Tumor-infiltrating leukocytes isolated as described above were first stained with antibodies against surface markers in MACS buffer. After washing, cells were resuspended in Annexin V binding buffer and stained with Annexin V FITC (SouthernBiotech, cat#10010-02). Alternatively, cells in MACS buffer were incubated with CellEvent Caspase-3/7 Green Detection Reagent (Thermo Fisher Scientific, cat#C10740) for 25 min at 37 °C, and then incubated with SYTOX AADvanced dead cell stain solution for 5 min at 37 °C. Stained samples were analyzed with a BD LSR II for Annexin V positive or Caspase-3/7 positive cells.

### TCF/LEF luciferase reporter assay

HEK293T cells were transfected with TCF/LEF firefly luciferase reporter, renilla luciferase control reporter (gifts from Dr. Xi He at Boston Children’s Hospital) and mammalian expression plasmids carrying *Lsd1*, *Tcf7* isoforms or *β-catenin*. Cells were lysed 48 h post-transfection and luciferase activity was measured and calculated according to the Dual-Luciferase Reporter Assay System (Promega) instruction manual.

### RNA extraction and real-time qPCR

For RNA extraction, cells were lysed by directly adding TRIzol (Life Technologies, cat#15596018) onto cells after supernatant removal. Total RNA extraction was performed according to the manufacturer’s instructions. The extracted RNA was reversely transcribed into cDNA using the PrimeScript™ RT Reagent Kit (TaKaRa, cat#RR037B) and used for real-time quantitative PCR (qPCR). SYBR green (Life Technologies, cat#A25743) and gene specific primers (listed in Supplementary Table 1) were used for PCR amplification and detection on a QuantStudio 3 real-time PCR system (Applied Biosystems). The qPCR data were normalized to Gapdh and presented as fold changes of gene expression in the test sample compared to the control.

### Gene deletion by CRISPR/Cas9

The guide RNA (gRNA) oligos targeting mouse *Lsd1* and *B2m* (sequences listed in Supplementary Table 1) were annealed and cloned into a lenti-CRISPR-v2-Puromycin^+^ vector (Addgene, cat# #52961), respectively. Lentivirus carrying lenti-CRISPR plasmid was prepared by co-transfecting HEK293T cells with four helper plasmids (pHDM-VSV-G, pHDM-tat1b, pHDM-HgPM2, and pRC-CMVRaII), followed by viral supernatant collection after 72 h. To delete *Lsd1*, MC38 cells were transduced with lenti-CRISPR virus with the addition of 8 μg/ml polybrene (Sigma-Aldrich, cat#H9268), and selected with 1 μg/ml puromycin for 2 days. Cells were serially diluted to allow clone generation from single cells. Clones were validated for *Lsd1* knockout by sequencing of target genomic regions and immunoblotting. To delete *B2m*, MC38 cells were selected with 1 μg/ml puromycin for 7 days after viral transduction. Cells were then stained with anti-B2m PE (Santa Cruz, cat#sc-32241 PE) and sorted on a BD Aria for B2m-negative cells.

### Co-immunoprecipitation

HEK293T cells were transfected with the indicated plasmids carrying HA- or Flag-tagged *Lsd1* or *Tcf7* isoforms, and harvested 48 h post-transfection. Cell pellets were lysed for 20 min on ice in IP lysis buffer (50 mM Tris pH 7.5, 150 mM NaCl, 0.1% NP-40, 0.1% Triton X-100, 10% glycerol) supplemented with Complete EDTA-free protease inhibitor cocktail (Sigma-Aldrich, cat#5892791001), 1 mM PMSF and PhosSTOP (Sigma-Aldrich, cat#04906837001). Protein lysates were briefly sonicated to shear chromatin and then cleared by 15 min centrifugation to pellet cell debris. Cleared protein lysates were incubated with anti-HA magnetic beads (Thermo Fisher Scientific, cat#88837) or anti-FLAG agarose beads (Sigma-Aldrich, cat#A2220) for 2 h at 4 °C. Immunoprecipitates were then washed three times with the IP lysis buffer and eluted using three resin volumes of the elution buffer (0.5 µg/ml FLAG peptide in IP lysis buffer for anti-FLAG resin; 1x SDS loading buffer for anti-HA resin).

### Immunoblotting analysis

Whole-cell lysates and immunoprecipitated eluents were denatured with SDS loading buffer and boiled for 5 min at 95 °C before resolved on SDS-PAGE gels. Proteins were then transferred onto nitrocellulose membrane, probed with the indicated primary antibodies and detected with HRP-conjugated secondary antibodies.

### RNA-seq sample processing

CD8^+^ TILs were isolated from MC38 tumor-bearing *Cd4-Cre*^*+*^*Lsd1*^*f/f*^ and *Lsd1*^*f/f*^ mice on day 18 as described above. CD8^+^ TILs from two individual tumor-carrying *Lsd1*^*f/f*^ mice were combined as a biological replicate, and CD8^+^ TILs from each individual tumor-carrying *Cd4-Cre*^*+*^*Lsd1*^*f/f*^ mouse was used as a biological replicate. CD8^+^ TILs were stained with antibodies against CD45.2 (clone 104), TCRβ (clone H57-587), CD8 (clone YTS156.7.7), CD44 (clone IM7) and PD-1 (clone 29F.1A12), and 7-AAD Viability Staining Solution was used to exclude dead cells. The CD45.2^+^TCRβ^+^CD8^+^CD44^+^PD-1^+^ cells were then sorted on a BD Aria. Approximately 10,000–20,000 sorted cells for each biological replicate were directly lysed in 1 ml TRIzol Reagent (Life Technologies, cat#15596018). After incubation for 5 min, 0.2 ml chloroform was added and mixed by inverting the tubes several times. Samples were incubated for 2–3 min and later centrifuged for 15 min at 12,000 × *g* at 4 °C. The upper aqueous phase containing the RNA was collected and mixed with an equal volume of 70% ethanol, which was then loaded into a spin column from a RNeasy Micro Kit (Qiagen, cat#74004) and subjected to RNA isolation according to the instruction manual. The on-column DNase digestion was conducted to eliminate DNA contamination.

Purified total RNA was quantified by Qubit (Invitrogen) and used to generate rRNA-depleted RNA with a NEBNext® rRNA Depletion Kit (New England Biolabs, cat#E6310S) according to the manufacturer’s instructions. The rRNA-depleted RNA was purified with a RNA Clean & Concentrator-5 kit (Zymo Research, cat#R1016) and then used to generate a directional RNA library with a NEBNext® Ultra™ II Directional RNA Library Prep Kit for Illumina® (New England Biolabs, cat#E7760L) and NEBNext® Multiplex Oligos for Illumina® (New England Biolabs, cat#E7335L) according to the manufacturer’s instructions. Library concentrations and quality were assessed on a Bioanalyzer and by qPCR. The library was sequenced at Nextseq 500 (Illumina) to generate reads from paired-ends (43 bp + 42 bp). The raw data are deposited at the Gene Expression Omnibus (GEO) under the subseries entry GSE147130.

### RNA-seq data analyses and functional interpretations

The software STAR^65^ (version 2.4.0e) was used to generate genome indices for mouse reference genome (GRCm38/mm10, December 2011), with two particular specifications including gene annotations, i.e., GENCODE^66^ (vM23, September 2019), and exon-exon junctions, i.e., 35 nucleotides used in constructing the splice junctions database. Next, the high quality paired-end RNA-seq reads were aligned to mouse reference genome, and the consequence of alignment served as the input for featureCounts (version 1.5.0)^67^ to quantify raw read counts for 55,385 annotated genes, including 21,856 protein-coding genes. Moreover, the normalized unit of reads per kilobase per million mapped reads (RPKM) was generated for every gene annotated in GENCODE, in order to fit the primary request of principle component analysis (PCA). The R function *prcomp* was used to perform PCA by using the genes having RPKM ≥ 1 in at least two samples, and the returned two vectors were used as coordinates to make a scatter plot in a 2-dimensional plane.

We used R package DESeq2^68^ (version 1.14.1) to identify differentially expressed (DEX) genes between WT and KO. The raw read count per gene served as the input for DESeq2. The GC content correction from CQN package was incorporated to DESeq2^69^. Since four samples were collected in either condition, they were treated as biological replicates to improve the reliability of DEX genes identification. Statistical tests for differential expression were based on a model using the negative binomial distribution. The reported statistical significances were corrected for multiple testing using the Benjamini–Hochberg procedure with a false discovery rate less than 0.01. In addition, to be called DEX genes we required the fold change > 1.5. The up- and downregulated genes in KO condition were separately queried to Gene Ontology Consortium for gene ontology enrichment assessment, including biological process (BP), molecular function (MF), and cellular component (CC). Moreover, we utilized the software GSEA (version 4.0.3)^70^ to survey the statistically significant concordant differences between KO and WT by using the normalized gene RPKM values, and consequently compared the KO upregulated gene sets to “C7: immunologic signatures” to explore any overrepresented functional term. In addition, a list of upregulated genes and a list of downregulated genes with the cutoff set at FC > 1.5 and FDR < 0.01 were sorted. The lists of these genes were uploaded onto the online DAVID (https://david.ncifcrf.gov/summary.jsp) bioinformatics resources to analyze for enriched GO terms under the category of GOTERM_BP_DIRECT.

### TCF1 ChIP-seq data analyses

The previously published TCF1 ChIP-seq data on splenic CD8^+^ T cells were downloaded from GEO under accession number GSE73240, for which call peaks were conducted with MACS v1.4.2 with TCF1 KO cells as control and with a stringent cutoff (FC ≥ 4, *p* < 10^−5^ and FDR < 0.05)^43^. 7807 TCF1 binding sites were retrieved and assigned to gene TSS (−2 kb to 2 kb).

### Statistical analyses

Statistical analyses were performed using GraphPad Prism 8 software and statistical significance was determined by *p* < 0.05. An unpaired Student’s *t* test was used for comparisons between two groups and a two-way ANOVA was used for multiple comparisons of tumor growth. For comparing mouse survival curves, a Log-rank (Mantel–Cox) test was used.

### Reporting summary

Further information on research design is available in the Nature Research Reporting Summary linked to this article.

## Supplementary information

Supplementary Information

Supplementary Data 1

Description of Additional Supplementary Files

Reporting Summary

## Data Availability

The RNA-seq data are deposited at the Gene Expression Omnibus (GEO) under the accession code GSE147130. The previously published ChIP-seq data are accessible with the code GSE73240. The remaining data of this study are available within the Article, Supplementary Information files or Source Data file. Source data are provided with this paper.

## Source data

Source data
