# Author Correction: LSD1 inhibition sustains T cell invigoration with a durable response to PD-1 blockade

**DOI:** 10.1038/s41467-021-27814-3

**Published:** 2022-01-05

**Authors:** Yi Liu, Brian Debo, Mingfeng Li, Zhennan Shi, Wanqiang Sheng, Yang Shi

**Affiliations:** 1 Division of Newborn Medicine and Epigenetics Program, Boston Children’s Hospital, Harvard Medical School, Boston, MA 02115 USA; 2 Ludwig Institute for Cancer Research, University of Oxford, Oxford, OX3 7DQ UK; 3 Department of Neuroscience and Kavli Institute for Neuroscience, Yale School of Medicine, New Haven, CT 06510 USA; 4 Institute of Immunology, and Department of Respiratory Disease of The First Affiliated Hospital, Zhejiang University School of Medicine, Hangzhou, Zhejiang, 310058 China

**Keywords:** Tumour immunology, Epigenetics

Correction to: *Nature Communications* 10.1038/s41467-021-27179-7, published online 24 November 2021.

The original version of this article contained errors in Fig. 1c, h, 3b and Supplementary Fig. 8c.

In Fig. 1c, h and Supplementary Fig. 8c, *p* values were incorrectly presented as 0.0016, 0.93, 0.0025, respectively. The correct *p* values are 0.0017 (Fig. 1c), 0.92 (Fig. 1h), 0.0013 (Supplementary Fig. 8c).

In Fig. 3b, the frequency of CD8 + T cells in the FTY720 group, day 20 flow cytometry panel was incorrectly presented as 0.0064. The correct value is 0.064.

The original Source data file contains the correct values.

These errors have been corrected in the PDF and HTML versions of the article and in the Supplementary Information file.

## Supplementary information

Updated Supplementary Information

