# Supplementary material for: LSD1 inhibition sustains T cell invigoration with a durable response to PD-1 blockade
Source: Nat Commun. 2021 Nov 24;12:6831. doi: 10.1038/s41467-021-27179-7 (PMC8613218; doi:10.1038/s41467-021-27179-7)
Supplement: Supplementary file 1 — Supplementary Information [file 41467_2021_27179_MOESM1_ESM.pdf]

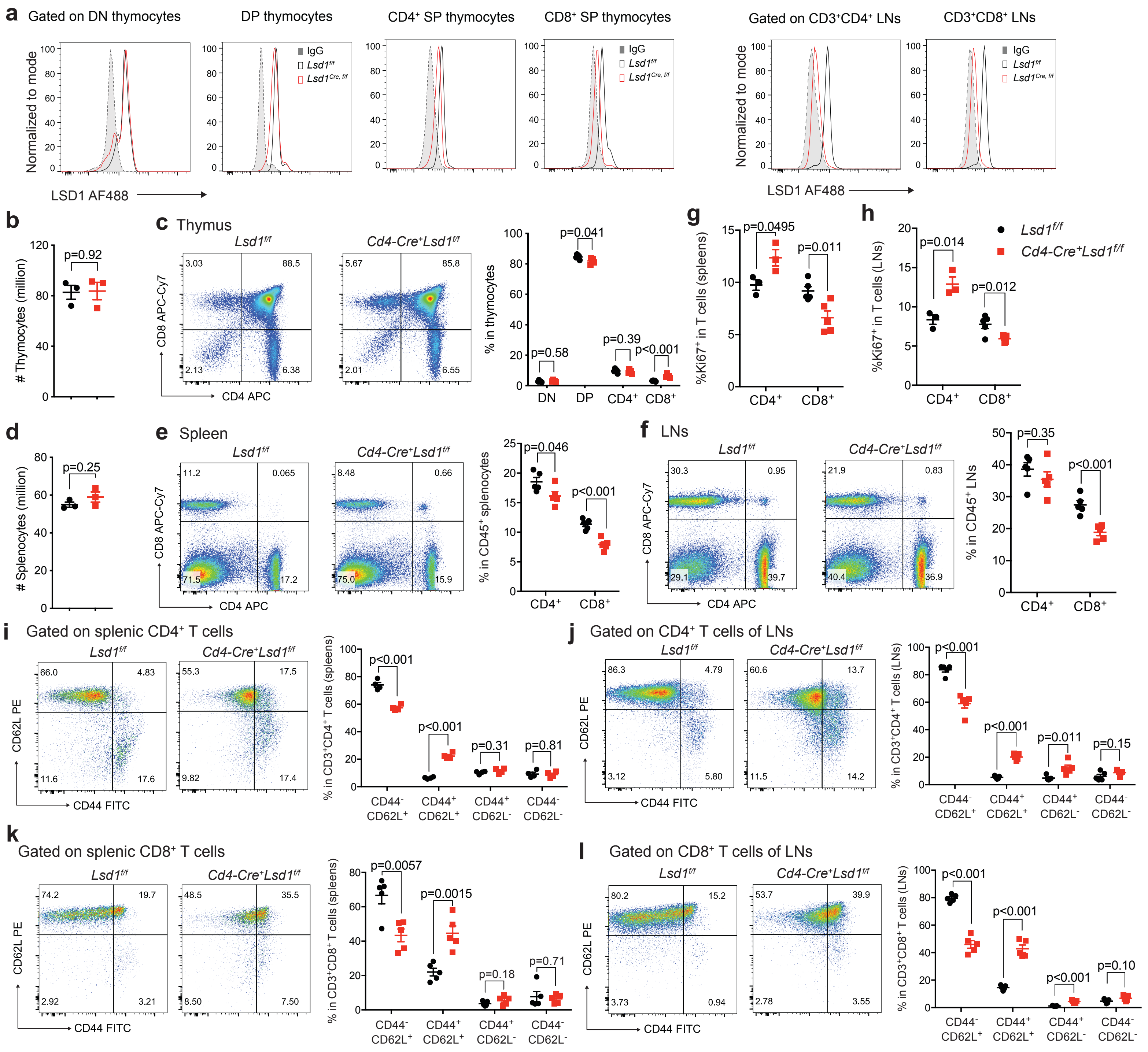

Supplementary Fig. 1 Characterization of T cells in T cell-specific *Lsd1* knockout mice.

a, Flow cytometry analysis of LSD1 protein expression in thymocytes at indicated developmental stages or in mature CD4$^+$ and CD8$^+$ T cells isolated from lymph nodes of *Lsd1*$^{f/f}$ and *Cd4-Cre*$^+$*Lsd1*$^{f/f}$ mice. **b, c**, Cell numbers of total thymocytes (**b**, n=3 per group) and percentages of thymocytes at different stages of T cell development (**c**, n=3 per group) analyzed by flow cytometry. **d, e**, Cell numbers of total splenocytes (**d**, n=3 per group) and frequencies of CD3$^+$CD4$^+$ and CD3$^+$CD8$^+$ T cells in spleens (**e**, n=5 per group). **f**, Frequencies of CD3$^+$CD4$^+$ and CD3$^+$CD8$^+$ T cells in lymph nodes (n=5 per group). **g**, **h**, Frequencies of Ki-67-expressing CD4$^+$ and CD8$^+$ T cells in spleens (**g**) and lymph nodes (**h**) (n=3 per group for CD4$^+$ and n=5 per group for CD8$^+$). **i-l**, Phenotypic analysis of CD4$^+$ (**i, j**) and CD8$^+$ (**k, l**) T cells in peripheral lymphoid organs based on the expression of surface markers CD44 and CD62L (n=5 per group). Data represent two independent experiments (**a-d**) or are pooled from two independent experiments (**e-l**), and are presented as mean ± SEM (**a-l**). Statistical significance was determined by two-sided unpaired t test (**a-l**).

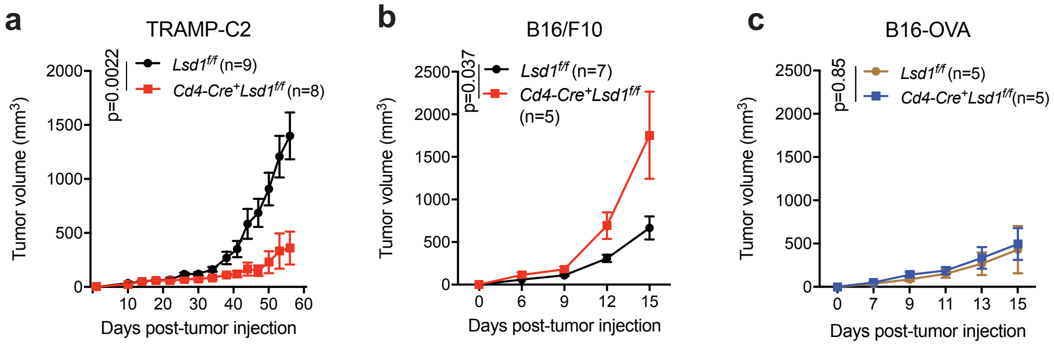

Supplementary Fig. 2 LSD1 depletion in T cells differentially affects tumor growth depending on tumor context.

a-c, Tumor growth curves of *Lsd1*$^{f/f}$ and *Cd4-Cre*$^{+}$*Lsd1*$^{f/f}$ mice subcutaneously inoculated with TRAMP-C2 (**a**), B16/F10 (**b**) or B16-OVA (**c**) tumor cells. Data represent two independent experiments (**a, b**) and are presented as mean ± SEM. Statistical significance was determined by two-sided unpaired t test.

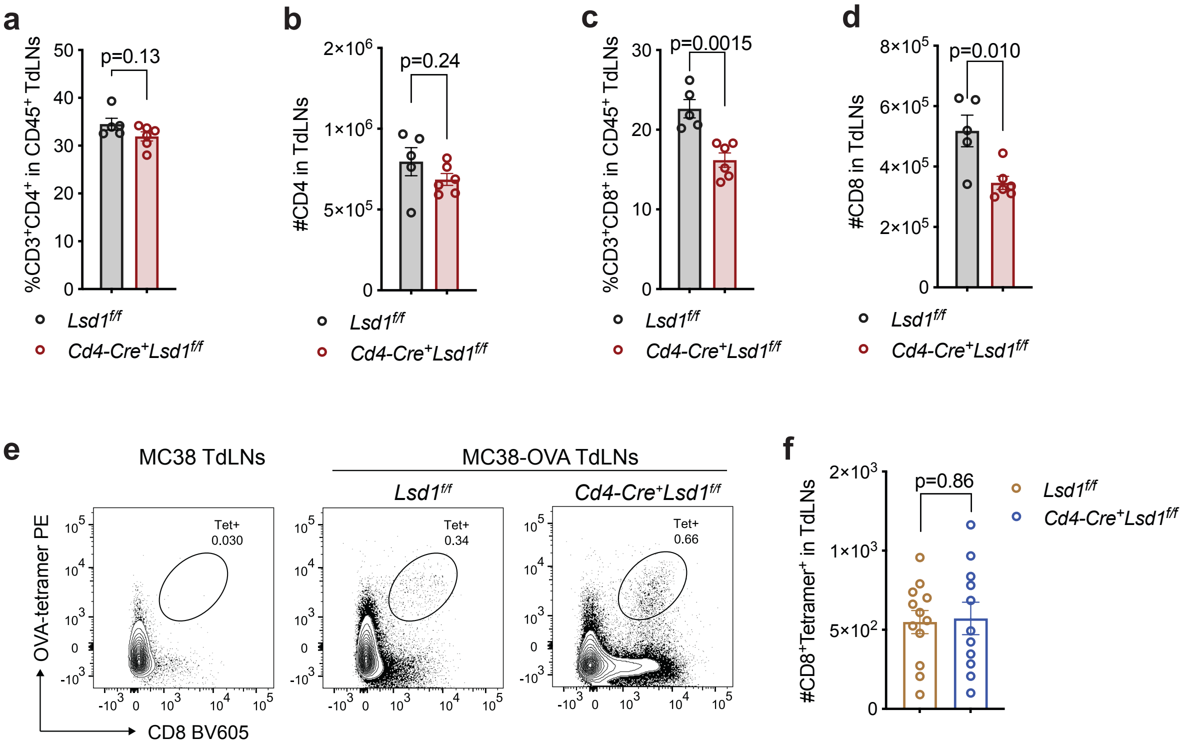

Supplementary Fig. 3 Cellularity of T cells in TdLNs.

a-**d**, Frequencies (**a**, **c**) and cell numbers (**b**, **d**) of CD4$^+$ and CD8$^+$ T cells in TdLNs of *Lsd1*$^{f/f}$ and *Cd4-Cre*$^+$*Lsd1*$^{f/f}$ mice carrying MC38 tumors (*Lsd1*$^{f/f}$ group, n=5; *Cd4-Cre*$^+$*Lsd1*$^{f/f}$ group, n=6). **e, f**, Representative flow plots (**e**) and cell numbers (**f**) of CD8$^+$Tetramer$^+$ T cells in TdLNs of *Lsd1*$^{f/f}$ and *Cd4-Cre*$^+$*Lsd1*$^{f/f}$ mice carrying MC38-OVA tumors (*Lsd1*$^{f/f}$ group, n=12; *Cd4-Cre*$^+$*Lsd1*$^{f/f}$ group, n=11). Data represent two independent experiments (**a-d**) or are pooled from three independent experiments (**f**), and are presented as mean ± SEM (**a**-**f**). Statistical significance was determined by two-sided unpaired t test (**a**-**f**).

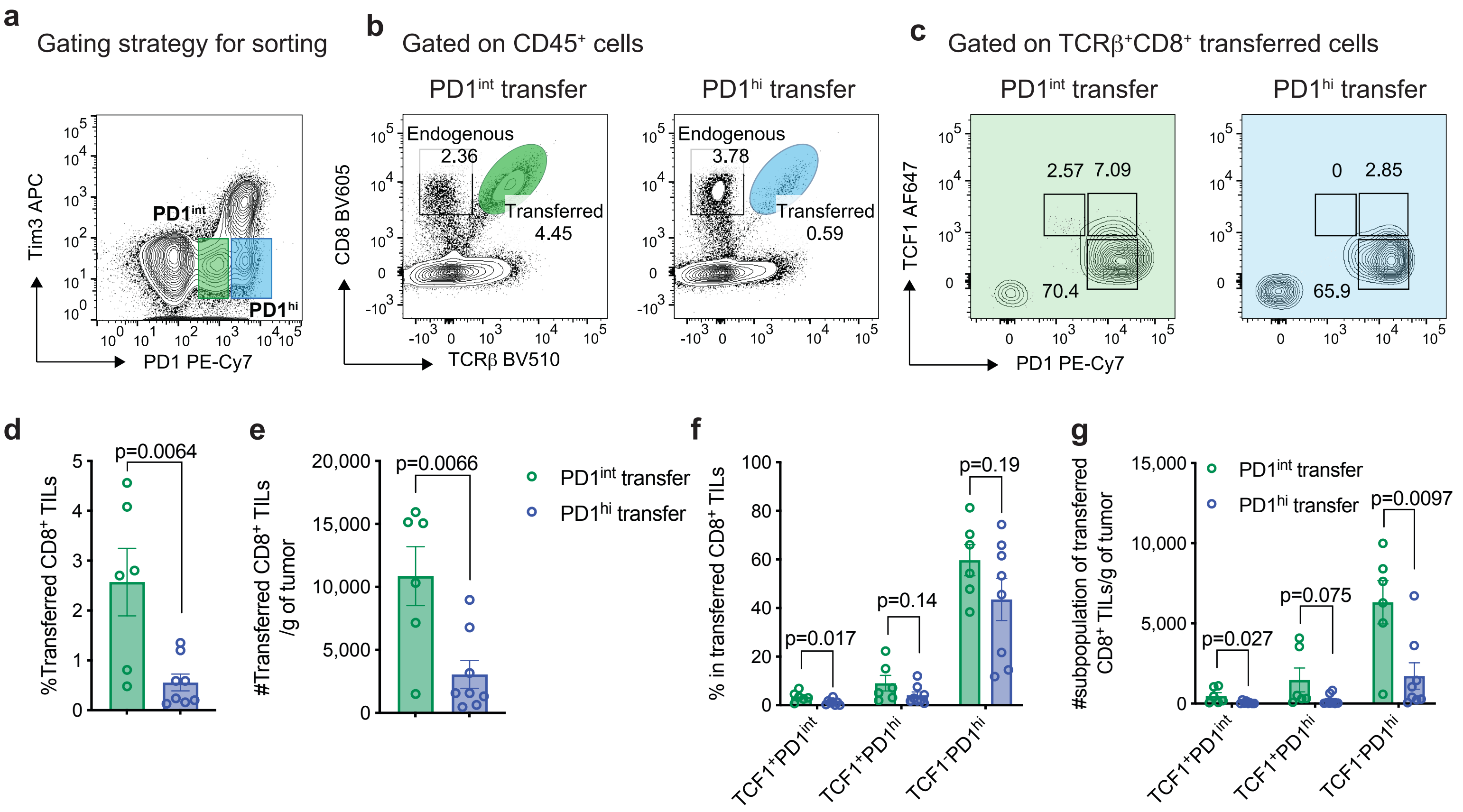

Supplementary Fig. 4 PD-1^{int} CD8$^+$ TILs generate numerically more terminally exhausted cells than do PD-1^{hi} cells.

a-c, Representative flow plots showing the gating strategy for sorting MC38 tumor-infiltrating PD-1^{int}Tim-3$^-$ (PD-1^{int}) and PD-1^{hi}Tim3$^-$ (PD-1^{hi}) CD8$^+$ T cells for adoptive cell transfer (**a**), the detection of donor CD8$^+$ TILs 12~14 days after being adoptively transferred into MC38 tumor-bearing recipient mice (**b**), and the phenotype of transferred cells (**c**). **d, e,** Frequencies (**d**) and cell numbers (**e**) of adoptively transferred CD8$^+$ TILs in the TME (PD1^{int} transfer group, n=6; PD1^{hi} transfer group, n=8). **f, g,** Frequencies (**f**) and cell numbers (**g**) of three subpopulations derived from adoptively transferred PD-1^{int} or PD-1^{hi} CD8$^+$ TILs (PD1^{int} transfer group, n=6; PD1^{hi} transfer group, n=8). Data are presented as mean ± SEM (**d-g**). Statistical significance was determined by two-sided unpaired t test (**d-g**).

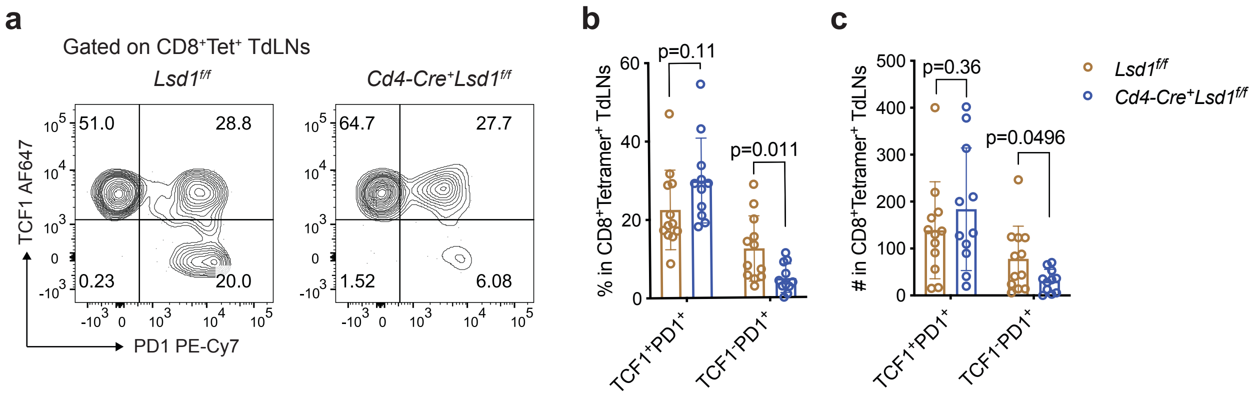

Supplementary Fig. 5 Phenotypic analysis of antigen-specific CD8⁺ T cells in TdLNs.
a-c, Representative flow plots (**a**), percentages (**b**) and cell numbers (**c**) of TCF1⁺PD-1⁺ and TCF1⁻PD-1⁺ cells in CD8⁺Tetramer⁺ TdLNs of mice carrying MC38-OVA tumors (*Lsd1$^{f/f}$* group, n=12; *Cd4-Cre⁺Lsd1$^{f/f}$* group, n=11). Data are pooled from three independent experiments and presented as mean ± SEM (**b, c**). Statistical significance was determined by two-sided unpaired t test (**b, c**).

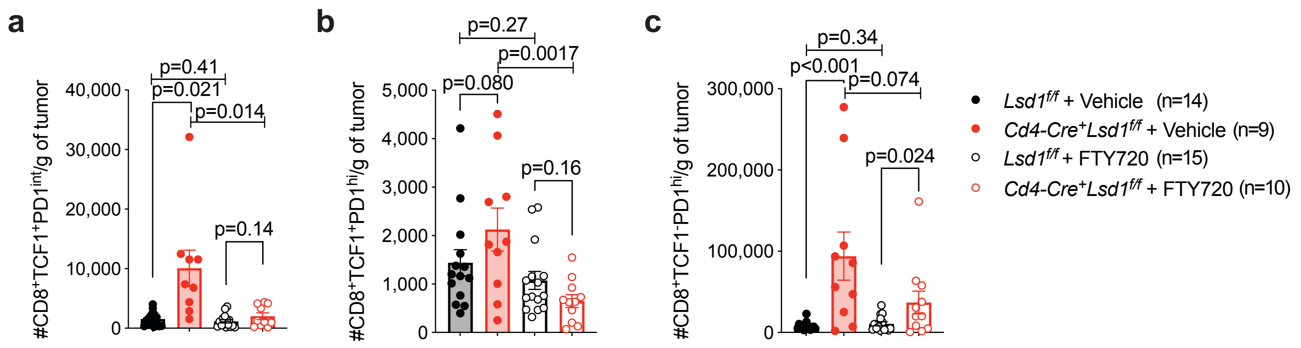

Supplementary Fig. 6 Phenotypic analysis of CD8$^+$ TILs in response to FTY720 treatment.

a-c, Cell numbers of TCF1$^+$PD-1^{int} (**a**), TCF1$^+$PD-1^{hi} (**b**) and TCF1$^-$PD-1^{hi} (**c**) subsets of CD8$^+$ TILs analyzed by flow cytometry on day 22 after MC38 tumor implantation. FTY720 treatment was initiated on day 12 and continued for 10 days. Data are pooled from two independent experiments and presented as mean ± SEM (**a**-**c**). Sample sizes are as indicated. Statistical significance was determined by two-sided unpaired t test (**a**-**c**).

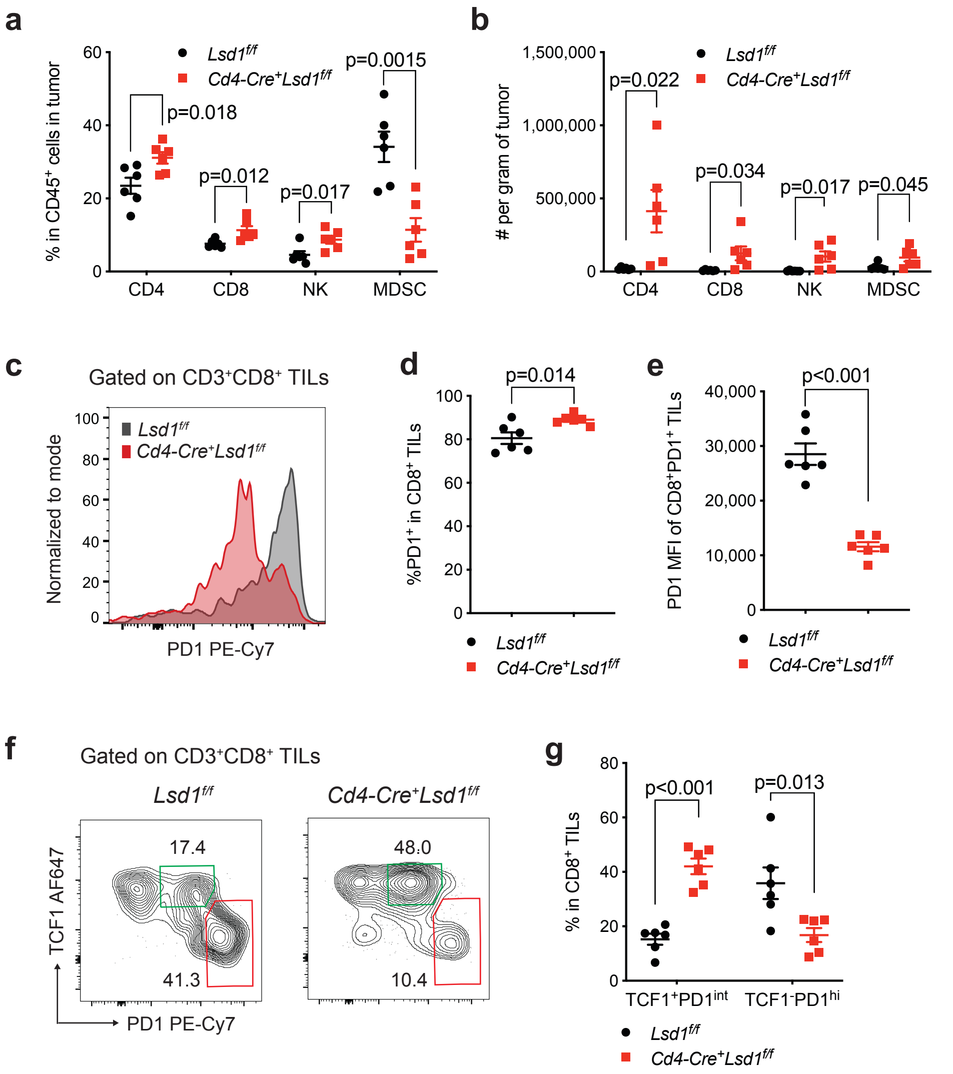

Supplementary Fig. 7 LSD1 depletion preserves TCF1⁺PD-1^{int} progenitor exhausted CD8⁺ T cells in TRAMP-C2 tumors.

a, **b**, Frequencies (**a**) and cell numbers (**b**) of intratumoral CD3$^+$CD4$^+$ T cells, CD3$^+$CD8$^+$ T cells, CD3$^-$CD49b$^+$ NK cells and CD11b$^+$Gr1$^+$ MDSCs among gated CD45$^+$ leukocytes analyzed by flow cytometry in *Lsd1$^{f/f}$* and *Cd4-Cre$^+$Lsd1$^{f/f}$* male mice carrying TRAMP-C2 tumors (n=6 per group). **c**-**e**, A representative flow plot of PD-1 expression by CD8$^+$ TILs (**c**), frequencies (**d**) and PD-1 MFI (**e**) of CD8$^+$PD-1$^+$ TILs (n=6 per group). **f**, **g**, Representative flow plots (**f**) and percentages (**g**) of TCF1$^+$PD-1^{int} and TCF1$^-$PD-1^{hi} cells in CD8$^+$ TILs (n=6 per group). Data represent two independent experiments and are presented as mean ± SEM (**a**-**g**). Statistical significance was determined by two-sided unpaired t test (**a**-**g**).

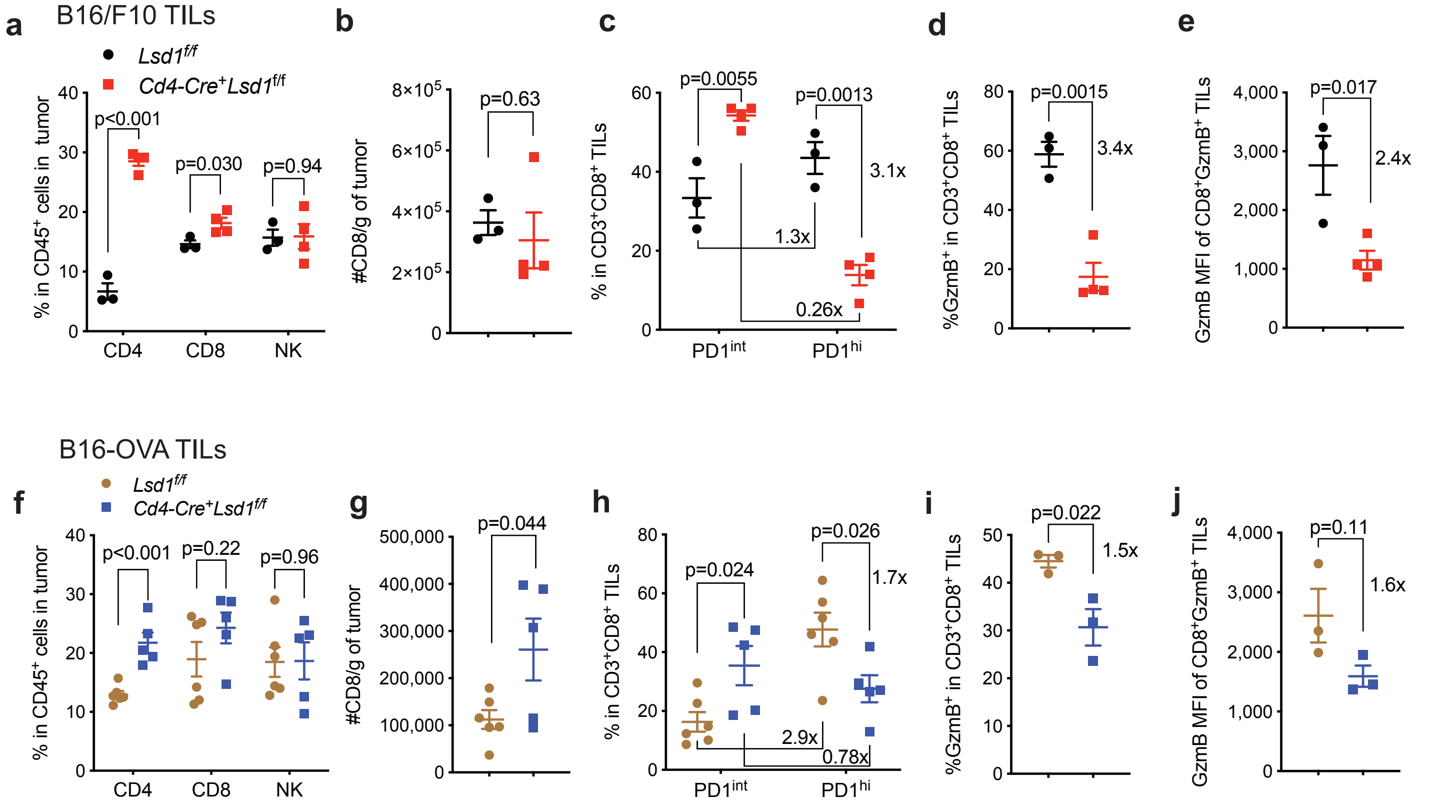

Supplementary Fig. 8 The PD-1^{int} progenitor subset of exhausted CD8$^+$ TILs are consistently elevated by LSD1 loss in B16 tumor models.

a-e, Percentages of CD4$^+$ T cells, CD8$^+$ T cells and NK cells among gated CD45$^+$ leukocytes (**a**), cell numbers of CD8$^+$ TILs (**b**), percentages of PD-1^{int} cells and PD-1^{hi} cells in CD8$^+$ TILs (**c**), percentages of GzmB$^+$ cells in CD8$^+$ TILs (**d**) and GzmB MFI of CD8$^+$GzmB$^+$ TILs (**e**), analyzed by flow cytometry on day 18 after B16/F10 tumor implantation (*Lsd1$^{f/f}$* group, n=3; *Cre$^+$Lsd1$^{f/f}$* group, n=4). **f-j,** Percentages of CD4$^+$ T cells, CD8$^+$ T cells and NK cells among gated CD45$^+$ leukocytes (**f**), cell numbers of CD8$^+$ TILs (**g**), percentages of PD-1^{int} cells and PD-1^{hi} cells in CD8$^+$ TILs (**h**), percentages of GzmB$^+$ cells in CD8$^+$ TILs (**i**) and GzmB MFI of CD8$^+$GzmB$^+$ TILs (**j**), analyzed by flow cytometry on day 18 after B16-OVA tumor implantation. n=6 (*Lsd1$^{f/f}$* group) or n=5 (*Cre$^+$Lsd1$^{f/f}$* group) over two independent experiments (**f-h**). n=3 per group (**i**, **j**). Data are presented as mean ± SEM (**a-j**). Statistical significance was determined by two-sided unpaired t test (**a-j**).

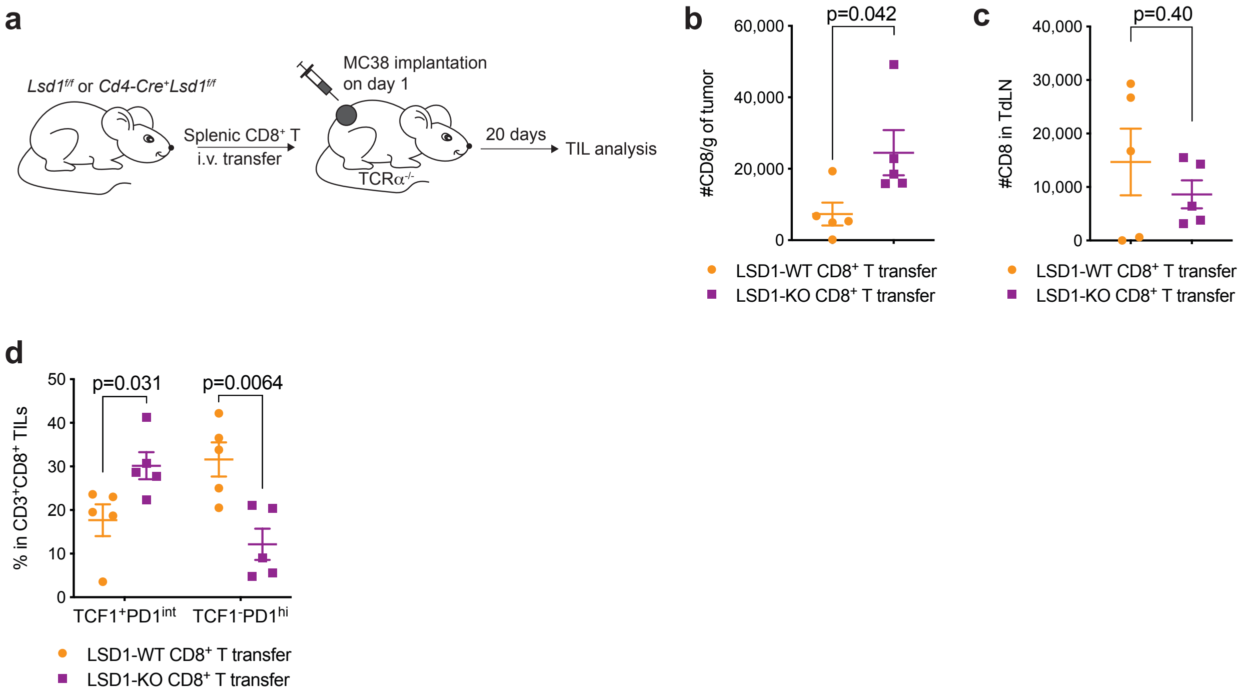

Supplementary Fig. 9 LSD1 depletion intrinsically enhances the infiltration of CD8+ TILs and their progenitor phenotype.

a, Experimental design of splenic CD8+ T cell transfer and tumor implantation. **b**, **c**, Cell numbers of transferred CD8+ T cells in MC38 tumors (**b**) or TdLNs (**c**). **d,** Percentages of TCF1+PD-1^{int} and TCF1-PD-1^{hi} cells in CD8+ TILs analyzed by flow cytometry. Data are presented as mean ± SEM (**b-d**, n=5 per group). Statistical significance was determined by two-sided unpaired t test (**b-d**).

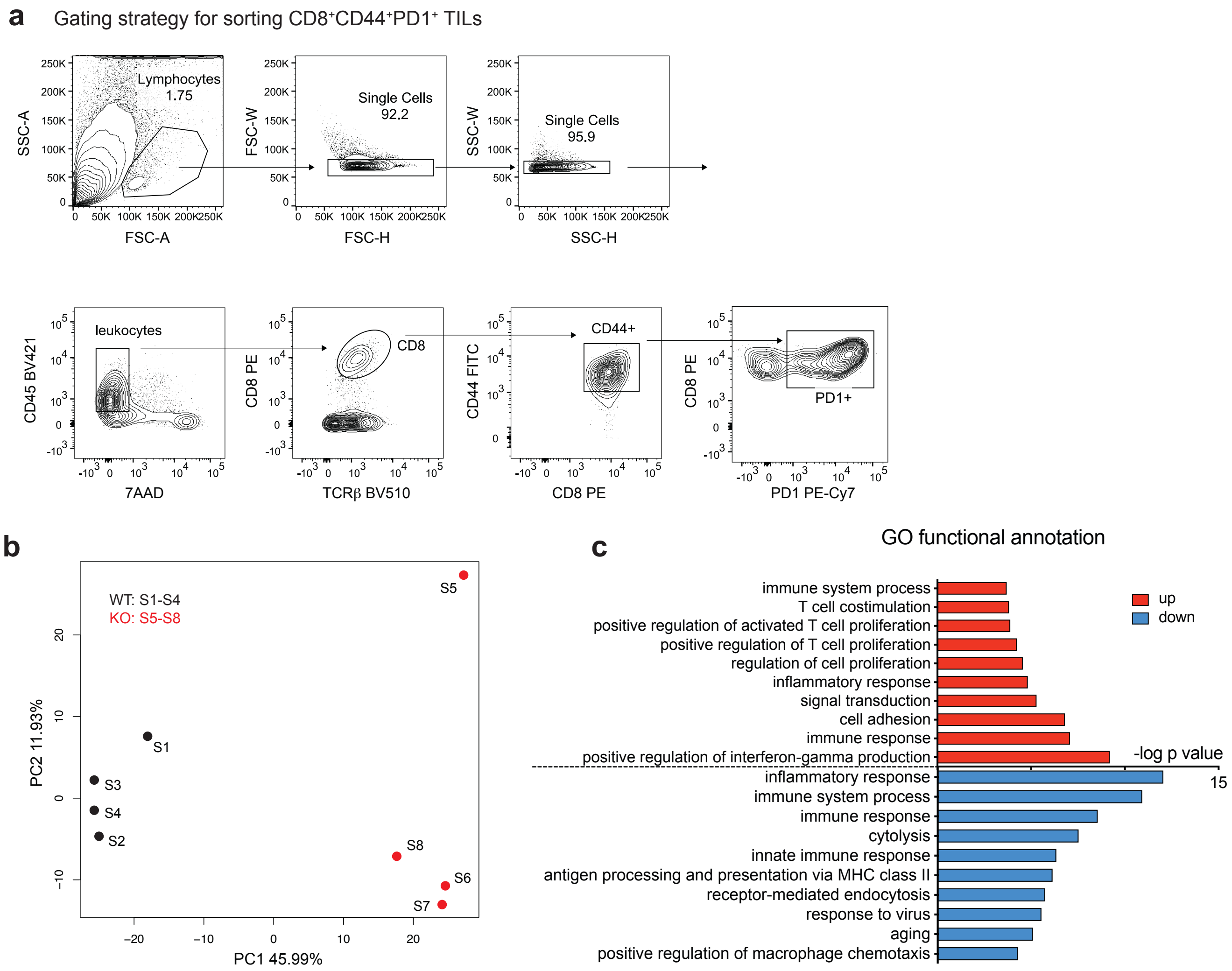

Supplementary Fig. 10 Biological processes regulated by LSD1 in CD8+ TILs.

a, Flow plots depicting the gating strategy for sorting CD8+CD44+PD-1+ TILs from MC38 tumors for RNA-seq analysis. **b**, Principal component analysis (PCA) of RNA-seq data on LSD1-deficient and wildtype CD8+CD44+PD-1+ TILs (n=4 per group). **c**, Gene ontology (GO) analysis of differentially expressed genes (FC > 1.5 and FDR < 0.01) in LSD1-deficient versus wildtype CD8+ TILs (n=4 per group).

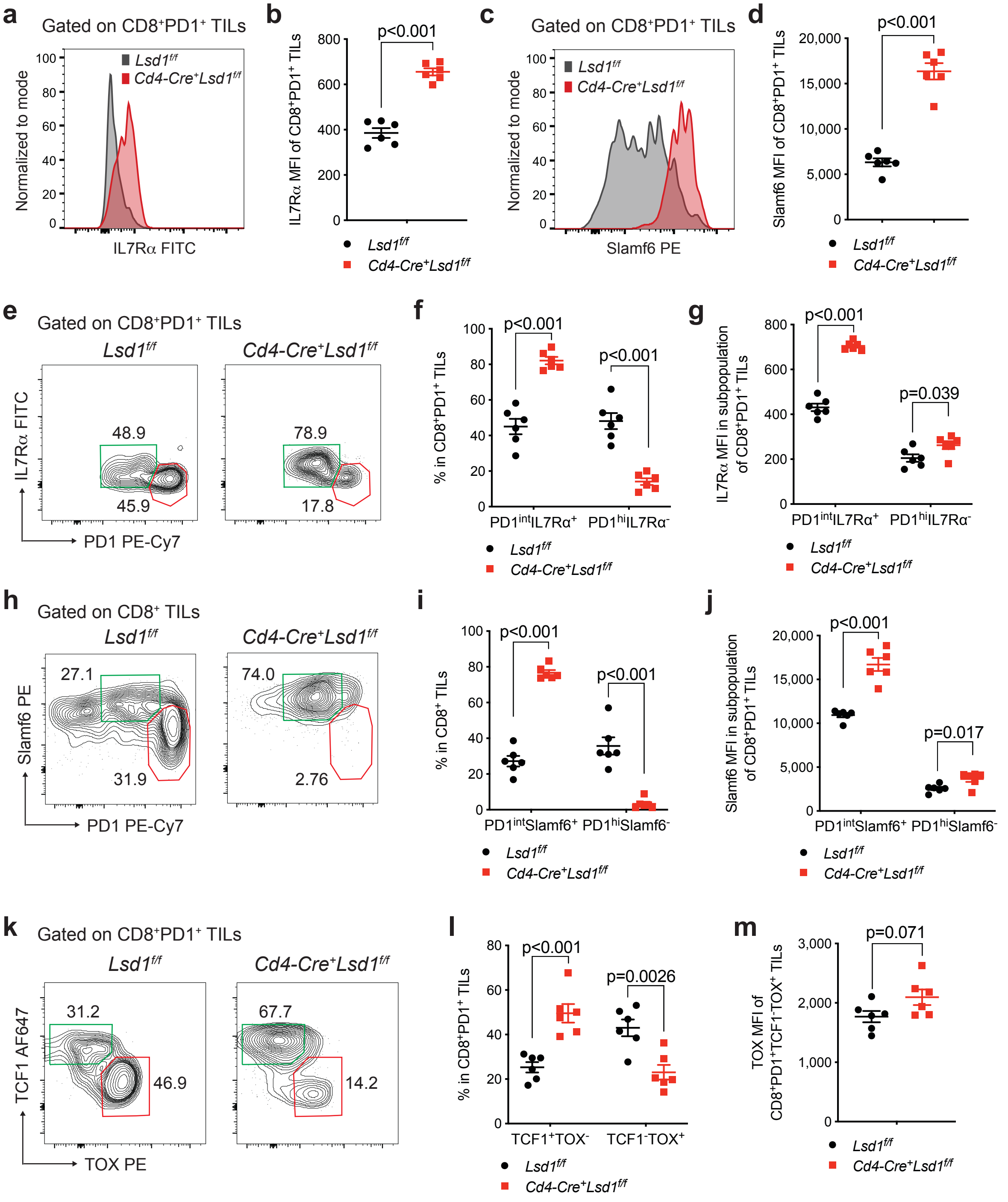

Supplementary Fig. 11 LSD1 loss upregulates self-renewal genes in progenitor exhausted CD8+ TILs of TRAMP-C2 tumors.

a, **b**, A representative flow plot of IL7Rα expression (**a**) and IL7Rα MFI (**b**) of CD8+PD-1+ TILs isolated from TRAMP-C2 tumors (n=6 per group). **c**, **d**, A representative flow plot of Slamf6 expression (**c**) and Slamf6 MFI (**d**) of CD8+PD-1+ TILs (n=6 per group). **e-g**, Representative flow plots (**e**), percentages (**f**) and IL7Rα MFI (**g**) of PD-1intIL7Rα+ and PD-1hiIL7Rα- cells in CD8+PD-1+ TILs (n=6 per group). **h-j**, Representative flow plots (**h**), percentages (**i**) and Slamf6 MFI (**j**) of PD-1intSlamf6+ and PD-1hiSlamf6- cells in CD8+ TILs (n=6 per group). **k**, **l**, Representative flow plots (**k**) and percentages (**l**) of TCF1+TOX- and TCF1-TOX+ cells in CD8+PD-1+ TILs (n=6 per group). **m**, TOX MFI of CD8+PD-1+TCF1-TOX+ TILs (n=6 per group). Data represent two independent experiments and are presented as mean ± SEM. Statistical significance was determined by two-sided unpaired t test (**a-m**).

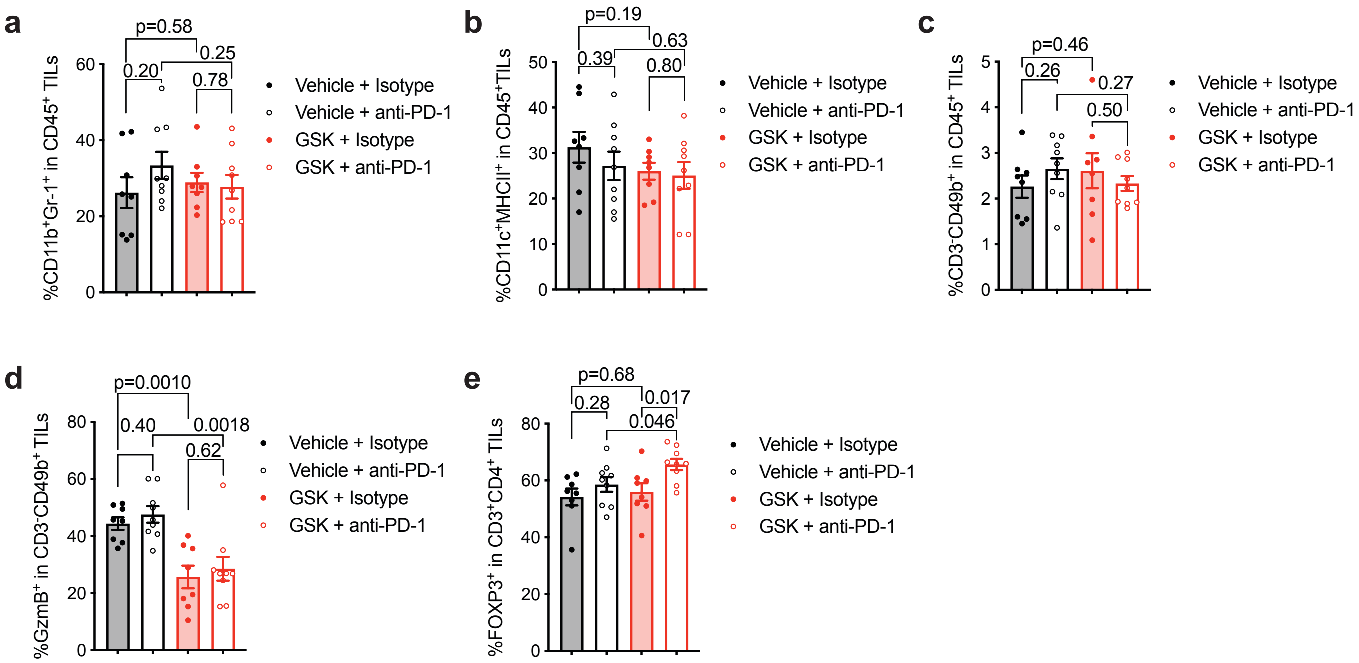

Supplementary Fig. 12 The effect of GSK2879552 on tumor-infiltrating immune cells.
a-c, Percentages of tumor infiltrating $CD11b^+Gr1^+$ MDSCs (**a**), $CD11c^+$MHC-II^+ DCs (**b**) and $CD3^-CD49b^+$ NK cells (**c**) among gated $CD45^+$ leukocytes analyzed by flow cytometry on day 18 after MC38 tumor implantation in wildtype mice receiving GSK2879552 treatment daily and/or anti-PD-1 treatment on day 14 and day 16. **d,** Percentages of $GzmB^+$ cells in tumor-infiltrating $CD3^-CD49b^+$ NK cells. **e,** Percentages of $FOXP3^+$ cells in $CD4^+$ TILs. Data represent two independent experiments and are presented as mean ± SEM (**a-e**). Vehicle + Isotype, n=8; Vehicle + anti-PD-1, n=9; GSK + Isotype, n=8; GSK + anti-PD-1, n=9 (**a-e**). Statistical significance was determined by two-sided unpaired t test (**a-e**).

Supplementary Table 1

RT-qPCR primer sequences		
Taget genes	Primer labels	Sequences
MuERV-L (MERVL)	MuERV-L-qPCR-F	TTTCTCAAGGCCCACCAATAGT (cited from Biol Reprod 68(2):651-4)
	MuERV-L-qPCR-R	GACACCTTTTTTAACTATGCGAGCT
mouse Line1	m-Line1-qPCR-F	TTTGGGACACAATGAAAGCA (cited from Nature 463, 237-240)
	m-Line1-qPCR-R	CTGCCGTCTACTCCTCTTGG
mouse IFN-α1	m-IFNα1-qPCR-F	CGGTGCTGAGCTACTGGC
	m-IFNα1-qPCR-R	TTTGTACCAGGAGTGTCAAGG
mouse IFN-β	m-IFNβ-qPCR-F	GGTGGAATGAGACTATTGTTG
	m-IFNβ-qPCR-R	AGGACATCTCCCACGTC
mouse ISG15	m-ISG15-qPCR-F1	GGTGTCCGTGACTAACTCCAT
	m-ISG15-qPCR-R1	TGGAAAGGGTAAGACCGTCCT
mouse OASL	m-OASL-qPCR-F1	CAGGAGCTGTACGGCTTCC
	m-OASL-qPCR-R1	CCTACCTTGAGTACCTTGAGCAC
mouse GAPDH	m-GAPDH-qPCR-F	TGACCTCAACTACATGGTCTACA
	m-GAPDH-qPCR-R	CTTCCCATTCTCGGCCTTG

CRISPR gRNA sequences		
Target genes	Sequences	Target location
mouse *Lsd1*	CCTGAGAGGTCATTCGGTCA	exon 3
mouse *B2m*	CTGGTGCTTGTCTCACTGAC	exon 1
