# Supplementary material for: LSD1 inhibition sustains T cell invigoration with a durable response to PD-1 blockade
Source: Nat Commun. 2021 Nov 24;12:6831. doi: 10.1038/s41467-021-27179-7 (PMC8613218; doi:10.1038/s41467-021-27179-7)
Supplement: Supplementary file 3 — Description of Additional Supplementary Files [file 41467_2021_27179_MOESM3_ESM.pdf]

Title: Supplementary Data 1:

Description: RNA-seq analysis showing differential gene expression of MC38 tumor-infiltrating $CD8^+CD44^+PD1^+$ T cells lacking LSD1 versus wildtype counterparts.
