# Supplementary material for: LSD1 inhibition sustains T cell invigoration with a durable response to PD-1 blockade
Source: Nat Commun. 2021 Nov 24;12:6831. doi: 10.1038/s41467-021-27179-7 (PMC8613218; doi:10.1038/s41467-021-27179-7)
Supplement: Supplementary file 4 — Reporting Summary [file 41467_2021_27179_MOESM4_ESM.pdf]

nature research

Corresponding author(s): Yang Shi, Wanqiang Sheng

Last updated by author(s): Oct 20, 2021

Reporting Summary

Nature Research wishes to improve the reproducibility of the work that we publish. This form provides structure for consistency and transparency in reporting. For further information on Nature Research policies, see Authors & Referees and the Editorial Policy Checklist .

Statistics

For all statistical analyses, confirm that the following items are present in the figure legend, table legend, main text, or Methods section.

n/a	Confirmed	
☐	☒	The exact sample size (*n*) for each experimental group/condition, given as a discrete number and unit of measurement
☐	☒	A statement on whether measurements were taken from distinct samples or whether the same sample was measured repeatedly
☐	☒	The statistical test(s) used AND whether they are one- or two-sided<br>*Only common tests should be described solely by name; describe more complex techniques in the Methods section.*
☐	☒	A description of all covariates tested
☒	☐	A description of any assumptions or corrections, such as tests of normality and adjustment for multiple comparisons
☐	☒	A full description of the statistical parameters including central tendency (e.g. means) or other basic estimates (e.g. regression coefficient) AND variation (e.g. standard deviation) or associated estimates of uncertainty (e.g. confidence intervals)
☐	☒	For null hypothesis testing, the test statistic (e.g. *F*, *t*, *r*) with confidence intervals, effect sizes, degrees of freedom and *P* value noted<br>*Give P values as exact values whenever suitable.*
☒	☐	For Bayesian analysis, information on the choice of priors and Markov chain Monte Carlo settings
☒	☐	For hierarchical and complex designs, identification of the appropriate level for tests and full reporting of outcomes
☒	☐	Estimates of effect sizes (e.g. Cohen's *d*, Pearson's *r*), indicating how they were calculated

Our web collection on statistics for biologists contains articles on many of the points above.

Software and code

Policy information about availability of computer code

Data collection

Flow data were acquired using FACSDiva 8.0.1 (BD Pharmingen). RNA-seq data were collected on a NextSeq500 (Illumina).

Data analysis

For RNA-seq:
The software STAR (version 2.4.0e) was used to generate genome indices for mouse reference genome (GRCm38/mm10, December 2011), with two particular specifications including gene annotations, i.e., GENCODE (vM23, September 2019), and exon-exon junctions, i.e., 35 nucleotides used in constructing the splice junctions database. Next, the high quality paired-end RNA-seq reads were aligned to mouse reference genome, and the consequence of alignment served as the input for featureCounts (version 1.5.0) to quantify raw read counts for 55,385 annotated genes, including 21,856 protein-coding genes. Moreover, the normalized unit of reads per kilobase per million mapped reads (RPKM) was generated for every gene annotated in GENCODE, in order to fit the primary request of principle component analysis (PCA). The R function prcomp was used to perform PCA by using the genes having RPKM ≥ 1 in at least two samples, and the returned two vectors were used as coordinates to make a scatter plot in a 2-dimensional plane.

We used R package DESeq2 (version 1.14.1) to identify differentially expressed (DEX) genes between WT and KO. The raw read count per gene served as the input for DESeq2. The GC content correction from CQN package was incorporated to DESeq2. Since four samples were collected in either condition, they were treated as biological replicates to improve the reliability of DEX genes identification. Statistical tests for differential expression were based on a model using the negative binomial distribution. The reported statistical significances were corrected for multiple testing using the Benjamini-Hochberg procedure with a false discovery rate less than 0.01. In addition, to be called DEX genes we required the fold change > 1.5. The up- and down- regulated genes in KO condition were separately queried to Gene Ontology Consortium for gene ontology enrichment assessment, including biological process (BP), molecular function (MF), and cellular component (CC). Moreover, we utilized the software GSEA (version 4.0.3) to survey the statistically significant concordant differences between KO and WT by using the normalized gene RPKM values, and consequently compared the KO up-regulated gene sets to "C7: immunologic signatures" to explore any overrepresented functional term.

For other data analysis:
Statistical analyses were performed using GraphPad Prism 8 software and statistical significance was determined by $p < 0.05$. An unpaired

Student's t test was used for comparisons between two groups and a two-way ANOVA was used for multiple comparisons of tumor growth. For comparing mouse survival curves, a Log-rank (Mantel-Cox) test was used.

For flow cytometry data:
FlowJo 10.4 is used for data analysis and graph generation.

For manuscripts utilizing custom algorithms or software that are central to the research but not yet described in published literature, software must be made available to editors/reviewers. We strongly encourage code deposition in a community repository (e.g. GitHub). See the Nature Research guidelines for submitting code & software for further information.

Data

Policy information about availability of data

All manuscripts must include a data availability statement. This statement should provide the following information, where applicable:
- Accession codes, unique identifiers, or web links for publicly available datasets
- A list of figures that have associated raw data
- A description of any restrictions on data availability

The RNA-seq data are deposited at the Gene Expression Omnibus (GEO) under the accession code GSE147130 (https://www.ncbi.nlm.nih.gov/geo/query/acc.cgi?acc=GSE147130). The previously published ChIP-seq data are accessible with the code GSE73240 (https://www.ncbi.nlm.nih.gov/geo/query/acc.cgi?acc=GSE73240).The remaining data of this study are available within the article, Supplementary information files or Source Data files (including raw data and uncropped blot scans).

Field-specific reporting

Please select the one below that is the best fit for your research. If you are not sure, read the appropriate sections before making your selection.

- [x] Life sciences
- [ ] Behavioural & social sciences
- [ ] Ecological, evolutionary & environmental sciences

For a reference copy of the document with all sections, see nature.com/documents/nr-reporting-summary-flat.pdf

Life sciences study design

All studies must disclose on these points even when the disclosure is negative.

Sample size	Group sizes for mouse experiments were determined empirically based upon prior knowledge of the intra-group variation of tumor growth and drug treatment. We used different sample size for different tumor models and different types of experiments, based previously published work (PMIDs: 33687985, 30778252, 28723893, 30559380, 30728504). For in vitro experiments (TCF1 Luciferase reporter assay, qPCR analysis), sample sizes were determined empirically based on previous publications or protocols (PMIDs: 33687985, 30894540).
Data exclusions	No data were exclcuded from the analyses.
Replication	Replicates were used in experiments as noted in figure legends . Experiments presented for which replication was attempted were successfully replicated.
Randomization	Age and sex-matched mice were used for in vivo experiments. Mice were randomized prior to any treatment. For other in vitro randomization (reporter assay, in vitro splenocytes stimulation etc), samples were randomized for transfection or inhibitor treatment and no bias was introduced.
Blinding	For experiments with animals, mice were randomly assigned to separate cages for different treatments or injections. The investigators were not blinded when performing the experiments for cage labeling and staffing needs. Since a number of procedures (including cell line injections, drug administrations, tumor measurements, tumor collections) were involved in the experiments, it is crucial to keep cage and animals properly labeled or tagged. It is not feasible to blind due to the complexity of the experiments and limited number of researchers. Even though blinding is technically impossible during many of the procedures, researchers were blinded during final step data collection and analysis. Each mouse was assigned an ID to ensure blinding and all analyses on the data was done in a blinded manner. The data acquiring process and analysis (including tumor measurements, flow cytometry analysis etc) is a consistent and objective process and no personal bias is introduced.

Reporting for specific materials, systems and methods

We require information from authors about some types of materials, experimental systems and methods used in many studies. Here, indicate whether each material, system or method listed is relevant to your study. If you are not sure if a list item applies to your research, read the appropriate section before selecting a response.

Materials & experimental systems

n/a	Involved in the study	
	x	Antibodies
	x	Eukaryotic cell lines
x		Palaeontology
	x	Animals and other organisms
x		Human research participants
x		Clinical data

Methods

n/a	Involved in the study	
x		ChIP-seq
	x	Flow cytometry
x		MRI-based neuroimaging

Antibodies

Antibodies used	SIINFEKL H-2Kb Tetramer (NIH Tetramer Core Facility); LIVE/DEAD™ Fixable Near-IR Dead Cell Stain Kit (ThermoFisher Scientific, L10119); IgG2a isotype control (clone 2A3, BioXCell, BE0089); anti-PD-1 (BioXCell, clone 29F.1A12, BE0273); CD45.2, BV421 (clone 104, BioLegend, Cat#109831); CD45.2, PE (clone 104, BioLegend, Cat#109807); CD45.1, FITC (clone A20, BioLegend, Cat#110705); CD3e, BV510 (clone145-2C11, BioLegend, Cat#100353); TCRb, BV510 (clone H57-587, BioLegend, Cat#109233); CD4, APC (clone RM4-5, BioLegend, Cat#100516); CD8b, APC/Cy7 (clone YTS156.7.7, BioLegend, Cat#126619); CD8b, PE (clone YTS156.7.7, BioLegend, Cat#126607); CD8a, BV605 (clone 53-6.7, BioLegend, Cat#100743); CD8a, BV510 (clone 53-6.7, BioLegend, Cat#100751); Foxp3, PE (clone FJK-16s, ThermoFisher Scientific, Cat#12-5773-82); Granzyme-B, FITC (clone GB11, BioLegend, Cat#515403); Ki-67, PercCP-Cy5.5 (clone B56, BD Biosciences, Cat#561284); CD44, FITC (clone IM7, BioLegend, Cat#103005); CD62L, BV510 (clone MEL-14, BioLegend, Cat#104441); CD62L, PE (clone MEL-14, BioLegend, Cat#104407); CD16/32 (clone 93, BioLegend, Cat#101320); 7-AAD viability staining solution (BioLegend, Cat#420404); CD11b, BV605 (clone M1/70, BioLegend, Cat#101237); Gr-1, APC-Cy7 (clone RB6-8C5, BioLegend, Cat#108423); TNFa, FITC (cloneMP6-XT22, BioLegend, Cat#506303); IL-2, PerCP-Cy5.5 (cloneJES6-5H4, BioLegend, Cat#503821); IFN-g, PE (clone XMG1.2, BioLegend, Cat#104407); PD-1, PE-Cy7 (29F.1A12, BioLegend, Cat#135215); Tim-3, PE(RMT3-23, BioLegend, Cat#119703); Tim-3, APC (RMT3-23, BioLegend, Cat#119705); TCF1/TCF7, AF647 (C63D9, CST, Cat#6709S); CD127, PE-Cy7 (A7R34, BioLegend, Cat#135013); CD127, FITC (A7R34, BioLegend, Cat#135007); TOX, PE (TXRX10, eBiosciences, Cat#12-6502-80); Slamf6, PE (clone 13G3, eBiosciences, Cat#12-1508-80).
Validation	Validation is present on the manufacturer's website as noted in the Methods section. All the information about the validation data can be found using the clone number, catalogue number and supplier name shown as above. Our flow cytometry data are also consistent with the data shown on the manufacturer's website.

Eukaryotic cell lines

Policy information about cell lines

Cell line source(s)	MC38 cell line was a gift from Arlene Sharpe, B16/F10 is a gift from David Fisher. Both cell lines were originally purchased from ATCC. MC38-Ova cell line was a gift from Ana Anderson. B16-OVA cell line was a gift from Nick Haining. HEK293T and TRAMP-C2 cell line were purchased from ATCC.
Authentication	None of these cell lines were authenticated after being received.
Mycoplasma contamination	All cell lines were confirmed negative for mycoplasma using a ATCC kit.
Commonly misidentified lines (See ICLAC register)	No commonly misidentified cell lines were used.

Animals and other organisms

Policy information about studies involving animals; ARRIVE guidelines recommended for reporting animal research

Laboratory animals	6~10-week old mice were used for all experiments. Wildtype C57BL/6 mice were purchased from The Jackson Laboratory. Cd4-Cre transgenic mice (purchased from The Jackson Laboratory, stock #017336) were crossed with Lsd1 floxed mice (gifts from Dr. Stuart Orkin at Boston Children's Hospital) to generate Cd4-Cre+Lsd1f/f knockout mice and Lsd1f/f littermate control mice. Immunodeficient TCRa knockout mice were originally purchased from The Jackson Laboratory (stock #002116) and bred in-house. CD45.1+ congenic mice (strain B6.SJL-PtprcaPepcb/BoyJ) were purchased from The Jackson Laboratory. Animals are housed with a 12 hr light/12hr dark cycle, 74 (+/-3) °F and 35-70% (+/-5%) humidity. Male animals were used for TRAMP-C2 related experiments. Female animals were mostly used for other experiments .
Wild animals	No wild animals were involved.
Field-collected samples	Study did not involve field-collected samples.
Ethics oversight	All animal procedures were performed in accordance with animal care guidelines and with the prior approval by the Boston Children's Hospital Institutional Animal Care and Use Committee.

Note that full information on the approval of the study protocol must also be provided in the manuscript.

Flow Cytometry

Plots

Confirm that:

☒ The axis labels state the marker and fluorochrome used (e.g. CD4-FITC).

☒ The axis scales are clearly visible. Include numbers along axes only for bottom left plot of group (a 'group' is an analysis of identical markers).

☒ All plots are contour plots with outliers or pseudocolor plots.

☒ A numerical value for number of cells or percentage (with statistics) is provided.

Methodology

Sample preparation	5x105 MC38, B16/F10, B16-OVA or 106 TRAMP-C2 cells were subcutaneously injected into the right flank of individual mouse and tumors were harvested on day 12~22 as indicated after tumor inoculation. Tumors were minced into small pieces and digested in RPMI1640 medium containing 400 U/ml type I collagenase (Worthington Biochemical Corporation, cat#LS004194) and 100 ug/ml DNase I (Sigma-Aldrich, cat#10104159001) for 20 ~ 30 mins at 37 °C. Digested tumor tissue samples were neutralized with R10 medium and then filtered through a 70 uM cell strainer to obtain single cell suspensions. Samples were pelleted and resuspended in 5 ml of 40% Percoll (GE Healthcare, cat#17-0891-01) and underlayed by 3 ml of 70 % Percoll in a 15 ml conical tube. After centrifugation at 2000 rpm for 20 mins with break set at 1, leukocytes were enriched at the interface between 40% and 70% Percoll gradient. Collected leukocytes from the gradient interface were then resuspended in ACK lysis buffer to remove red blood cells and then stained with antibodies against surface markers and intracellular proteins, or SIINFEKL H-2Kb Tetramer (NIH Tetramer Core Facility) as needed. For cytokine staining, leukocytes were first stimulated with 1 uM Ova257-264 peptides (Anaspec, cat#AS-60193-1) or PMA/Ionomycin in the presence of Golgiplug for 4 hours.
Instrument	Stained tumor infiltrating leukocyte (TIL) samples were run on a BD LSR II, FACSymphony or sorted on a BD Aria.
Software	Flow data were acquired by FACSDIVA 8.0.1 (BD Pharmingen) and analyzed by FlowJo 10.4.1.
Cell population abundance	All sorts had a purity > 95%, checked by post-sort re-sampling.
Gating strategy	Gating strategy was summarized in Supplementary Fig. 10a and gates were drawn based on single-stain and unstained control.

☒ Tick this box to confirm that a figure exemplifying the gating strategy is provided in the Supplementary Information.
